# 8 Å structure of the outer rings of the *Xenopus laevis* nuclear pore complex obtained by cryo-EM and AI

**DOI:** 10.1007/s13238-021-00895-y

**Published:** 2022-01-11

**Authors:** Linhua Tai, Yun Zhu, He Ren, Xiaojun Huang, Chuanmao Zhang, Fei Sun

**Affiliations:** 1 National Key Laboratory of Biomacromolecules, Institute of Biophysics, CAS Center for Excellence in Biomacromolecules, Chinese Academy of Sciences, Beijing, 100101 China; 2 The Ministry of Education Key Laboratory of Cell Proliferation and Differentiation and the State Key Laboratory of Membrane Biology, College of Life Sciences, Peking University, Beijing, 100871 China; 3 Center for Biological Imaging, Institute of Biophysics, Chinese Academy of Sciences, Beijing, 100101 China; 4 University of Chinese Academy of Sciences, Beijing, 100049 China; 5 Bioland Laboratory (Guangzhou Regenerative Medicine and Health Guangdong Laboratory), Guangzhou, 510005 China

**Keywords:** nuclear pore complex, cryo-EM, *Xenopus laevis*, AlphaFold2, nuclear ring, cytoplasmic ring

## Abstract

The nuclear pore complex (NPC), one of the largest protein complexes in eukaryotes, serves as a physical gate to regulate nucleocytoplasmic transport. Here, we determined the 8 Å resolution cryo-electron microscopic (cryo-EM) structure of the outer rings containing nuclear ring (NR) and cytoplasmic ring (CR) from the *Xenopus laevis* NPC, with local resolutions reaching 4.9 Å. With the aid of AlphaFold2, we managed to build a pseudoatomic model of the outer rings, including the Y complexes and flanking components. In this most comprehensive and accurate model of outer rings to date, the almost complete Y complex structure exhibits much tighter interaction in the hub region. In addition to two copies of Y complexes, each asymmetric subunit in CR contains five copies of Nup358, two copies of the Nup214 complex, two copies of Nup205 and one copy of newly identified Nup93, while that in NR contains one copy of Nup205, one copy of ELYS and one copy of Nup93. These in-depth structural features represent a great advance in understanding the assembly of NPCs.

**Supplementary Information:**

The online version contains supplementary material available at 10.1007/s13238-021-00895-y.

## Introduction

In eukaryotes, a double-layered membrane encloses a large organelle called the nucleus to separate genetic materials from the cytoplasm (Lin and Hoelz, 2019). The nuclear pore complex (NPC) fuses the inner and outer nucleus membrane (INM and ONM) to form the sole gateway mediating cargo transfer between the nucleoplasm and cytoplasm (Hoelz et al., 2011; Hampoelz et al., 2019). NPCs are formed by approximately 30 different kinds of nucleoporins (Nups) with multiple copies in an eightfold symmetrical assembly. Each NPC contains approximately 550 proteins in fungi or approximately 1000 proteins in vertebrates (Rout et al., 2000; Cronshaw et al., 2002; Ori et al., 2013). NPCs have a cylindrical appearance throughout their overall organization, where a central channel mediating bidirectional nucleocytoplasmic cargo transfer exists (Hinshaw et al., 1992; Akey and Radermacher, 1993). Three scaffold rings, including an inner ring (IR), a cytoplasmic ring (CR) and a nuclear ring (NR), anchored onto the nuclear envelope (NE), provide bases for other functional parts, such as the cytoplasmic filament, nuclear basket, and permeability barrier (Beck et al., 2004; Beck et al., 2007; Maimon et al., 2012; Bui et al., 2013).

Detailed structural information is necessary for a mechanistic understanding of NPC functions, but it has long been hindered by the enormous size and high dynamicity of NPCs. Cryo-electron tomography (cryo-ET) along with subtomogram averaging (STA) has been applied to reach ~2 nm resolution of NPC structures from multiple species, including *Homo sapiens* (*H. sapiens*), *Xenopus laevis* (*X. laevis*)*, Chlamydomonas reinharadtii* (*C. reinharadtii*), *Schizosaccharomyces pombe* (*S. pombe*) and *Saccharomyces cerevisiae* (*S. cerevisiae*) (Bui et al., 2013; Appen et al., 2015; Kim et al., 2018; Mosalaganti et al., 2018; Allegretti et al., 2020; Zhang et al., 2020; Zimmerli, 2020). Based on these studies, the basic architectures of CR, IR and NR have been solved by rigid docking of crystal structures of several Nups (Lin et al., 2016; Lin and Hoelz, 2019), such as the model reported by Alexander et al. in 2015 (PDB 5A9Q, aliased as the 2015-model) (Appen et al., 2015) and Lin et al. in 2016 (aliased as the 2016-model) (Lin et al., 2016). In these scaffold rings, eight asymmetric units (or subunits) lay in a head-to-tail fashion to form the backbones. In both CR and NR, named the outer rings, the backbones in each subunit are formed by one or two Y-shaped complexes, also known as the Nup84 complex in fungi or the Nup107-160 complex in vertebrates (Hsia et al., 2007; Kampmann and Blobel, 2009; Seo et al., 2009; Bui et al., 2013; Kelley et al., 2015). In the Y complex of vertebrates, Nup85, Nup43 and Seh1 form the short arm region, Nup160 and Nup37 form the long arm region, and Sec13, Nup96, Nup107 and Nup133 form the stem region (Appen et al., 2015).

As major members of NPC scaffold rings, the outer rings of CR and NR are essential for building and maintaining NPC structures. CR provides docking sites for cytoplasmic filaments to regulate importin α/β-dependent nucleocytoplasmic transport and messenger ribonucleoprotein (mRNP) export (Bernad et al., 2004; Hutten and Kehlenbach, 2006). NR provides docking sites for the transportation of factors and cargos through the nuclear channel similar to preribosomes and mRNPs (Delavoie et al., 2019) and stabilizes the nuclear basket substructure on the nucleoplasm side of the NPC. In addition to the Y complex scaffold, the CR and NR have several other components, such as the Nup358 complex and Nup214 complex in CR, and ELYS (embryonic large molecule derived from the yolk sac, also known as Mel-28 or AHCTF1) in NR. Recently, cryo-electron microscopic (cryo-EM) single particle analysis (SPA) has been applied to study the detailed architecture of *X. laevis* NPC CR and reached 5.5 Å for the most rigid part of CR (aliased as the 2020-model) (Huang et al., 2020), yet the reconstruction suffered from anisotropic resolution caused by the preferred orientation problem. For NR, due to the lack of a high-resolution structure, protein components were still recognized only at the domain level.

In this study, we used modified cryo-EM SPA approaches to collect data not only for NPCs on a flattened NE with stage tilting but also for the side-view NPCs on the edge of folded NE. Then, we determined the significantly improved cryo-EM map of the CR and NR of the *X. laevis* NPC with an isotropic resolution up to 8 Å and local resolution reaching 4.9 Å. Meanwhile, with the aid of the recently emerged highly accurate protein structure prediction tool AlphaFold2 (Jumper et al., 2021), we managed to build the most complete pseudoatomic model of the CR and NR to date, including Y complexes and multiple flanking components. With the significantly improved model, we identified novel structural features of Nups in the CR and NR, including tight interactions in the Y complex hub mediated by the Nup160 C-terminal domain (CTD), fruitful interactions between Nup205 and surrounding Nups, five copies of Nup358 wrapped around the CR subunit stem region, two copies of Nup214 complexes attached to the CR subunit short arm region, detailed interactions between ELYS and Nup160 in NR, and the existence of Nup93 bridging the stems of Y complexes in both CR and NR. Overall, our results improve the understanding of the detailed assembly and functions of NPC outer rings.

## Results

### Structure determination of the outer ring subunit of *x. laevis* NPCs

Considering that the NPCs on a flattened NE showed severe preferred orientation problems yielding an anisotropic resolution of the cryo-EM map, we used an improved sample-collection strategy according to previous structural studies for *X. laevis* NPCs (Eibauer et al., 2015). Briefly, in addition to the data collection with the stage tilting angle set from 30 to 60 degrees, NPCs on the edge of a folded NE, which are shown in the side-view orientation, were especially selected and imaged (Figs. S1 and S2). Application of this approach significantly improved Fourier space sampling and resulted in cryo-EM reconstructions with nearly isotropic resolution (Figs. S3 and S4). Three local masks were applied to each CR or NR subunit to improve the map qualities of local regions. For CR, the highest resolution reached 8 Å for the core region of the CR subunit with a local resolution reaching 7.2 Å, while for the whole CR subunit and CR Nup358 region, the resolution reached 8.7 Å and 8.9 Å, respectively (Fig. S3). For NR, the final average resolutions were 8.1 Å for the NR subunit region, 7.8 Å for the NR core region and 8.6 Å for the NR Nup133 region (Fig. S4), where the highest local resolution reached was 4.9 Å for the most rigid part of the NR subunit. According to the three-dimensional Fourier shell correlation (3D-FSC) (Tan et al., 2017), all six reconstructions have sphericity scores better than 0.9 (0.91 to 0.95), suggesting no significant anisotropy in the reconstructions (Figs. S3 and S4).

**Figure 1:**
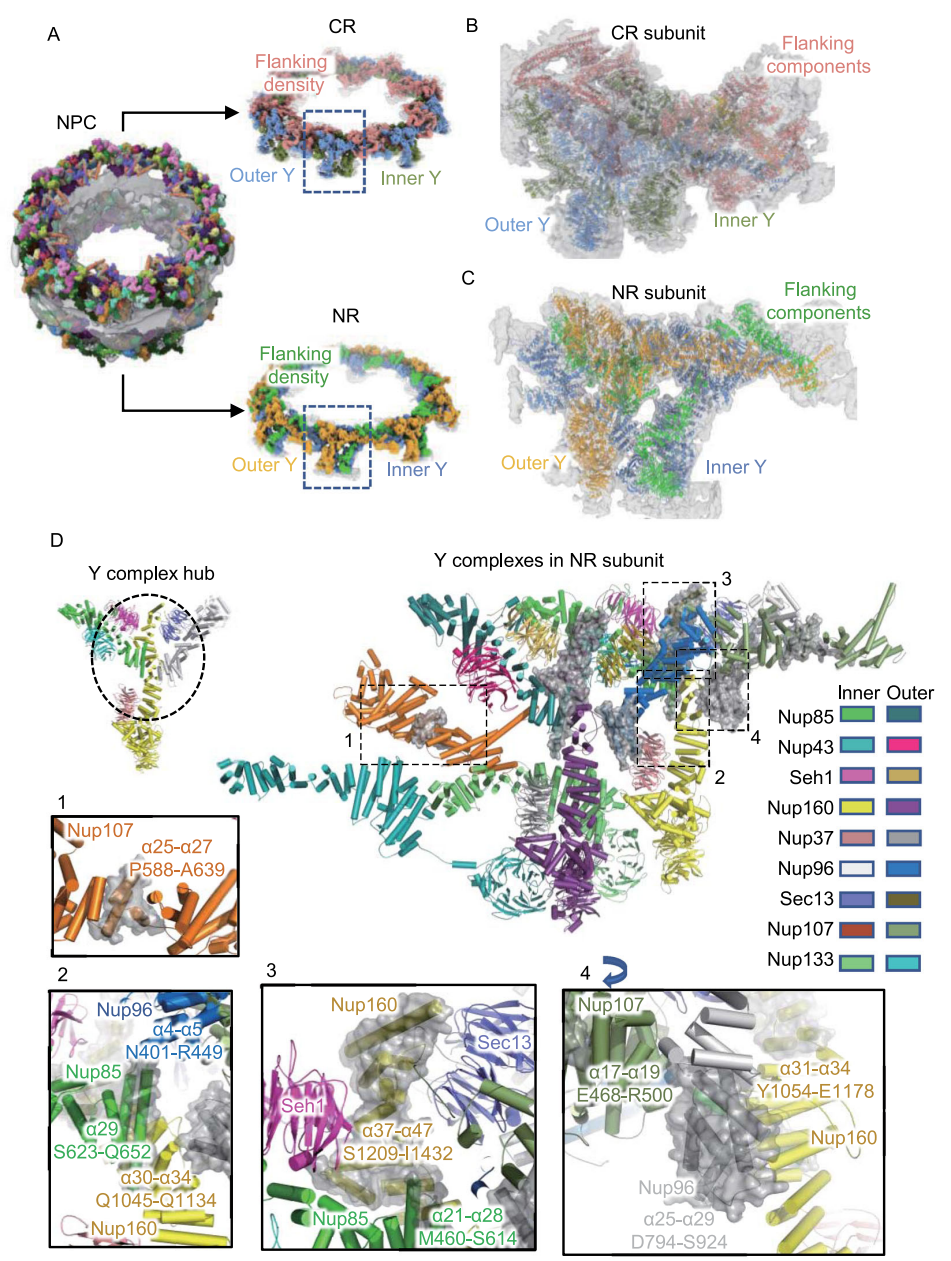
The pseudoatomic model of the CR and NR from the ***X. laevis*** oocyte NPC. (A) Overall view of the *X. laevis* NPC CR and NR structure, displaying the inner and outer Y complexes in each asymmetric unit, as well as the densities other than the Y complexes. (B) Model-map overlay of the NPC CR subunit. The map density is displayed in gray with transparency. Models of the inner Y complex, outer Y complex and extra densities are in the same colors as in (A). (C) Model-map overlay of the NPC NR subunit. The map density is displayed in gray with transparency. Models of the inner Y complex, outer Y complex and extra densities are in the same colors as in (A). (D) Model display of two Y complexes in the NR subunit. All Nups are colored differently and labeled. The major differences compared with the previous 2015-model are highlighted in the transparent gray surface. Nup107 region and Y complex hub regions are shown in zoomed-in views. The 4th zoomed-in view is rotated for better display. Important regions are labeled with the sequence and secondary structure numbers

By using AlphaFold2 (Jumper et al., 2021), we predicted highly accurate protein structures of all NPC Nups from *X. laevis* or *Xenopus tropicalis* (*X. tropicalis*). Then, with the help of molecular dynamics flexible fitting (MDFF) refinement and manual adjustment, we successfully modeled 32 components into each CR subunit (Fig. 1A and 1B) and 21 components into each NR subunit (Fig. 1A and 1C) with reliable secondary structure details (Figs. S5 and S6) (Movies S1–S3). It is worth noting that the refined model of each outer ring component against its corresponding local map is highly consistent with the model predicted by AlphaFold2, and only slight bending or domain shifts were found in a few models (Figs. S7 and S8), indicating that the structural predictions were highly accurate (Jumper et al., 2021). The overall resolution estimated by model-to-map FSC reached 9 Å for NR and 9.3 Å for CR, similar to the resolution estimated from gold standard Fourier shell correlation (FSC) criteria (Figs. S3D and S4D), further confirming that no significant resolution anisotropy exists in our reconstructions (Tan et al., 2017). In our model of CR, there are 22,372 residues in each subunit, which extends 102% compared with the CR of the 2016-model (11,064 residues) and 52.4% compared with the CR of the 2020-model (14,683 residues) (Lin et al., 2016; Huang et al., 2020). In our model of NR, a total of 15664 residues were built in each subunit, so we extended the number of residues by approximately 82.6% compared with the NR of the 2015-model (8,578 residues) and by approximately 41.6% compared with the NR of the 2016-model (11,064 residues) (Appen et al., 2015; Lin et al., 2016). Since the structural features of β-propeller domains among Y complex Nups have been well established, these extensions are mainly located in the α-helical regions (Hoelz et al., 2011; Lin and Hoelz, 2019).

### The CTDs of Nup85, Nup160 and Nup96 stabilize the Y complex hub

In the outer rings, two Y complexes lay in parallel, with a slight shift relative to one another, to form the backbone of the asymmetric unit (Fig. 1A). Most Nups of the Y complex have been well studied in both fungi and vertebrates. Nup85, Seh1 and Nup43 form the short arm of the Y complex; Nup160 and Nup37 form the long arm; and Sec13, Nup96, Nup107 and Nup133 form the stem. The stem base covers Sec13, Nup96 and the N-terminal domain (NTD) of Nup107 and the stem tip covers the C-terminus of Nup107 and Nup133 (Fig. 1D) (Bui et al., 2013; Appen et al., 2015). Two domain invasion motifs (DIMs) exist in Nup85-Seh1 and Nup96-Sec13 to form complete β-propeller structures (Hsia et al., 2007; Brohawn et al., 2008; Debler et al., 2008). These typical structures and features are all confirmed in our map and models (Figs. S5 and S6).

In our most complete CR and NR model from the *X. laevis* NPC, we identified several new features in the Y complex. In addition to the completion of the previously broken Nup107 structure, we clarified the important interaction hub connecting the two arms and stem base involving the CTDs of Nup85, Nup160 and Nup96 (Fig. 1D). For Nup85, we modeled a total of 29 α-helices, and S623-Q652 forms the last long α-helix, which makes contact with Nup96 (α4-α5, N401-R449) and Nup160 (α30-α34, Q1045-Q1134) (Fig. 1D) (Lin et al., 2016). For Nup160, compared with 36 previously identified α-helices in the 2016-model, we extended it to 47α-helices until reaching the C-terminus. The α37-α47 helices at the Nup160 CTD (S1209-I1432) form the core structure of the Y complex hub by making contact with Seh1, Nup85 (α21-α28, M460-S614) and Sec13 (Fig. 1D). For Nup96, which starts with the DIM in Sec13, we modeled all 29 α-helices in total, with the last 5 α-helices (D794-S924) making contact with Nup107 (α17-α19, E468-R500) and Nup160 (α31-α34, Y1054-E1178) (Fig. 1D). Overall, Nup85, Nup160 and Nup96 come together at their CTDs, forming a hub structure to maintain the overall Y-shaped structure.

The pseudoatomic model of the CR subunit was also built on our cryo-EM map, and the aforementioned interaction pattern of the Y complex hub was also confirmed. Thus, we compared the models of CR and NR and found that in addition to the flanking components unique for each ring, such as Nup358 or Nup214 complex for CR and ELYS for NR, their Y complex scaffold shares very similar architecture. The root-mean-square error (RMSD) value for double Y complexes in CR and NR is 4.6 Å (Fig. S9), and no large shifts are found for all the domains in Y complex Nups. For individual Y complexes, the RMSD values for inner Y and outer Y in CR, inner Y and outer Y in NR, and the inner Y in CR and NR, the outer Y in CR and NR are 5.6 Å, 6.2 Å, 4.8 Å and 3.8 Å, respectively (Fig. S9). The only significant differences are found in the comparison of inner and outer Y complexes, where the inner Nup133 has a shift of ~8 nm at the CTD. This shift should be related to the shorter circumference for the eight inner Y complexes than the outer Y complexes when forming a concentric ring of CR and NR. The consistency of the scaffold structure in CR and NR agrees well with previous reports (Bui et al., 2013; Appen et al., 2015; Lin et al., 2016; Kosinski et al., 2016). Moreover, it is worth noting that the local density map for the NTD of Nup160 in CR exhibits a lower local resolution than that in NR (Figs. S3 and S4), indicating the larger dynamics for the long arm region of the Y complex in CR. This kind of dynamic of CR may be due to two reasons. First, under certain conditions, such as energy depletion, constriction may occur in the CR region of NPCs (Zimmerli, 2020). Second, CR has only 32 β-propeller domains (16 from Nup160 and 16 from Nup133) anchored onto the NE, while NR has 8 or 16 more (from ELYS) to enhance the stability of Y complexes onto the membrane and increase the local stability of the Nup160 NTD (Appen et al., 2015).

### Five copies of Nup358 reside in each CR subunit

**Figure 2:**
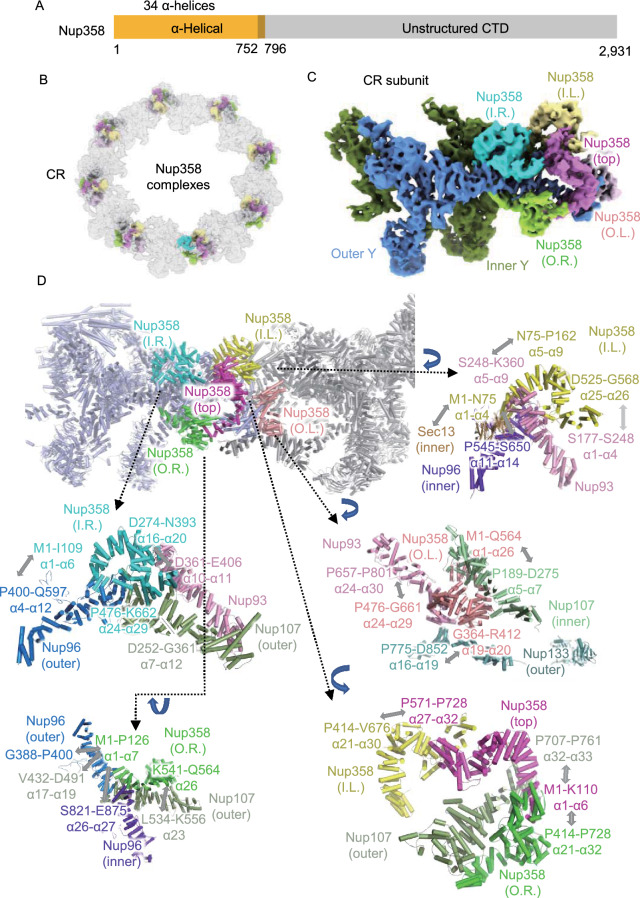
The structures and interaction details of five copies of Nup358 in each CR subunit. (A) Domain assignment of modeled part of Nup358. (B) Location of 40 copies of Nup358 in CR, while the 5 copies of Nup358 in each subunit are colored differently. (C) Location of 5 copies of Nup358 in the CR subunit, colored differently. (D) Interactions of 5 copies of Nup358 with surrounding Nups. Some zoomed-in views are rotated for better display

Nup358, as the largest Nup in vertebrates, plays an essential role in the biological functions of NPC through its multiple domains. Nup358 contains an α helical region in the NTD (Fig. 2A), followed by multiple domains separated by unstructured regions, including the Ran-binding domain, zinc finger domain, E3 ligase domain and cyclophilin domain (Wu et al., 1995; Kassube et al., 2012; Lin et al., 2013; Lin and Hoelz, 2019; Huang et al., 2020). Recent studies showed that the density corresponding to Nup358 looks like several clamps near the stems of Y complexes (Wu et al., 1995; Appen et al., 2015), and the copy number of Nup358 in each CR subunit may be 2 or 4 (Kassube et al., 2012; Huang et al., 2020). By using AlphaFold2, we predicted the structure of the Nup358 NTD, and found that this clamp-shaped structure could fit well in the local density of a previously assigned location for Nup358 (Fig. S5I**).** Strikingly, a total of 5 copies of Nup358 NTD could be well modeled into this region, suggesting that there should be at least 5 Nup358 proteins stably bound to the stem region of Y complexes in each CR subunit (Fig. 2B and 2C). The identification of 5 copies of Nup358 in each CR subunit could also be confirmed by the well-fitting of our CR model into the reported NPC structure from HeLa cells (Fig. S10) (Appen et al., 2015).

To distinguish each Nup358, we named them inner-left (I.L.), inner-right (I.R.), outer-left (O.L.), outer-right (O.R.) and top one according to their spatial locations, assuming the observer stands inside the nuclear channel (Fig. 2D). The two outer Nup358 proteins are located farther from the nuclear channel than the inner ones, while the top Nup358 is situated on top of the other four copies. These Nup358 proteins have rich contacts with surrounding CR Nups in different ways. Taking inner-right Nup358 as an example, its α1 to α6 helices (M1 to I109) interact with the α4 to α12 helices (P400 to Q597) of outer Nup96, α16 to α20 helices (D274 to N393) interact with the α10 to α11 helices (D361 to E406) of Nup93, and the α24 to α29 helices (P476 to K662) interact with the α7 to α12 helices (D252 to G361) of outer Nup107 (Fig. 2D). The other four Nup358 have similar rich interactions (Fig. 2D). Briefly, outer-right Nup358 binds to outer Nup107 and inner/outer Nup96; inner-left Nup358 binds to Sec13, inner Nup96 and Nup93; outer-left Nup358 binds to inner Nup107, outer Nup133 and Nup93; top Nup358 binds to outer Nup107, inner-left Nup358 and outer-right Nup358. Overall, the top Nup358 seems to act as a lid to cover the remaining Nup358 proteins, and the latter form direct and extensive interactions with inner and outer Y complexes to stabilize the stem region. The anchoring of these Nup358 NTDs onto CR will facilitate other domains of Nup358 or other Nup358 related proteins to function in physiological nucleocytoplasmic transport.

### Two Nup214 complexes lay in parallel in each CR subunit

**Figure 3:**
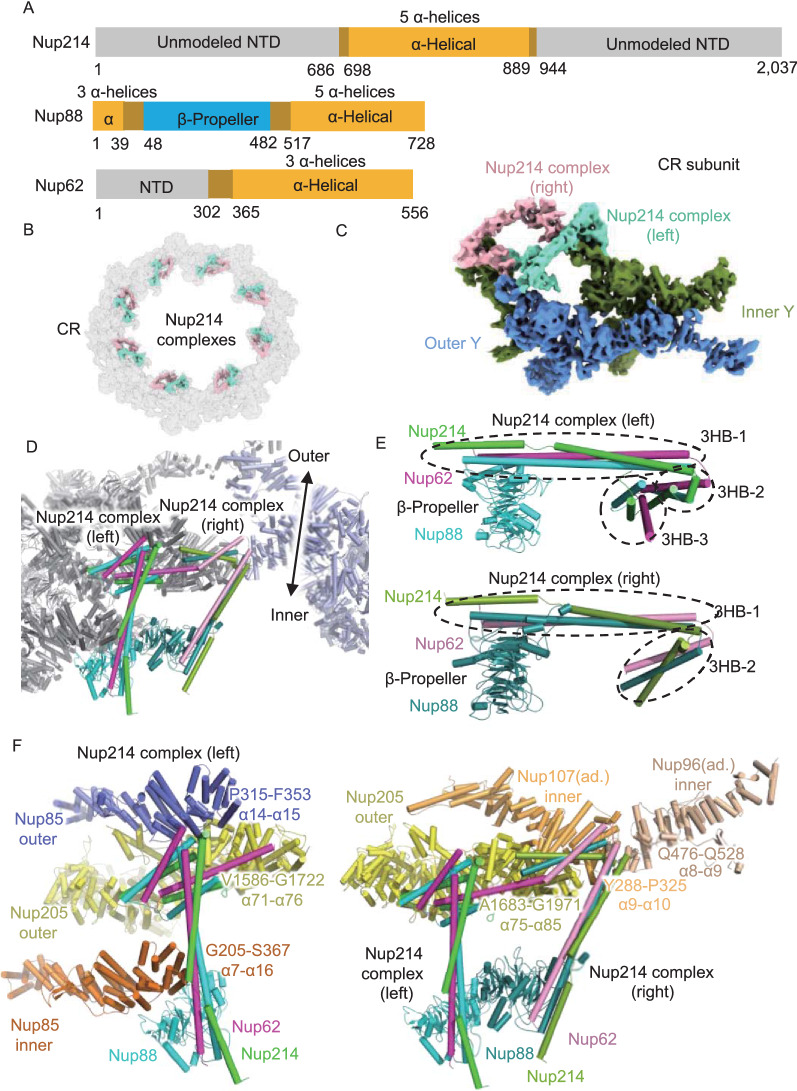
The structures and interaction details of two copies of Nup214 complexes in each CR subunit. (A) Domain assignment of modeled parts of Nup214, Nup88 and Nup62. (B) Location of 16 copies of the Nup214 complex in CR. The Nup214 complex in each subunit is colored differently. (C) Location of 2 copies of the Nup214 complex in the CR subunit. The left Nup214 complex is colored in chartreuse, and the right Nup214 complex is colored in light pink. (D) Model of the left and right Nup214 complex attached to two CR subunits. (E) Domain display of left and right Nup214 complexes. (F) Interaction sites of left and right Nup214 complexes

As another major component of the cytoplasmic filament, the Nup214 complex is believed to form an mRNP export platform on the cytoplasmic face of NPCs and coordinate the mRNP remodeling process to ensure the unidirectional transportation process (Gaik et al., 2015; Fernandez-Martinez et al., 2016). However, the structural details of the Nup214 complex remain elusive to date, including the exact copy number, its relative locations to the Y complex and the pseudoatomic model (Huang et al., 2020). The Nup214 complex is regarded to have at least three major components: Nup214, Nup88 and Nup62 in vertebrates (Fig. 3A) or Nup159, Nup82 and Nsp1 in fungi. It was reported that the Nup159 complex form a P-shaped homodimer configuration (Gaik et al., 2015; Fernandez-Martinez et al., 2016; Allegretti et al., 2020), but this kind of structure was not found in the CR structure of *X. laevis* (Huang et al., 2020).

Using AlphaFold2, we predicted the complex structure of Nup214, Nup88 and Nup62 and found that these three Nups could form a rake-shaped conformation (Fig. S5J), including a β-propeller domain as the handle, a helix bundle as the body and two helix bundles as the head. Then, we fitted this rake-shaped structure into the flanking density above the short arms of two Y complexes and found that there should be two copies of Nup214 complexes in each CR subunit (Fig. 3B and 3C). To distinguish these two complexes, we named them the left and right complexes according to their spatial locations, assuming that the observer stands inside the nuclear channel (Fig. 3D). In the left Nup214 complex, the rake handle is made up of the β-propeller domain of Nup88, while the unusually long rake body is made up of three helices bundle (3HB) from Nup214, Nup88 and Nup62, named as 3HB-1. For the rake head, there are two 3HB structures, 3HB-2 and 3HB-3, while every helix in the 3HBs comes from different Nups. The right Nup214 complex has basically the same conformation as the left complex, except for the missing 3HB-3 domain (Fig. 3E).

With this much improved model of these two Nup214 complexes in the CR subunit, we found several important interaction features. For the left Nup214 complex, the β-propeller of Nup88 and the 3HB-1 domain bind to the α7 to α16 helices (G205 to S367) of inner Nup85. Then, 3HB-2 and 3HB-3 make close contact with the α14 to α15 helices (P315 to F353) of outer Nup85, and the α71 to α76 helices (V1586 to G1722) of outer Nup205 (Fig. 3F). For the right Nup214 complex, the β-propeller of Nup88 binds to the left Nup214 complex, its 3HB-1 and 3HB-2 domains connect with α75 to α85 helices (A1683 to G1971) of the outer Nup205, α9 to α10 helices (Y288 to P325) of the inner Nup107 in adjacent CR subunit, and α8 to α9 helices (Q476 to Q528) of the inner Nup96 in the adjacent CR subunit (Fig. 3F). It seems that the two Nup214 complexes help to stabilize the two short arm regions of Y complexes in the CR subunit and contribute to the head-to-tail fashion of adjacent CR subunits. Moreover, the NTDs of both left and right Nup214 complexes point to the nuclear channel, allowing for the correct formation of the mRNP export platform to coordinate the proper mRNP remodeling process at the cytoplasmic end of the nuclear channel (Napetschnig et al., 2007; Napetschnig et al., 2009; Roloff et al., 2013; Fernandez-Martinez et al., 2016).

### The ELYS NTD interacts with four adjacent Nups in the NR subunit

**Figure 4:**
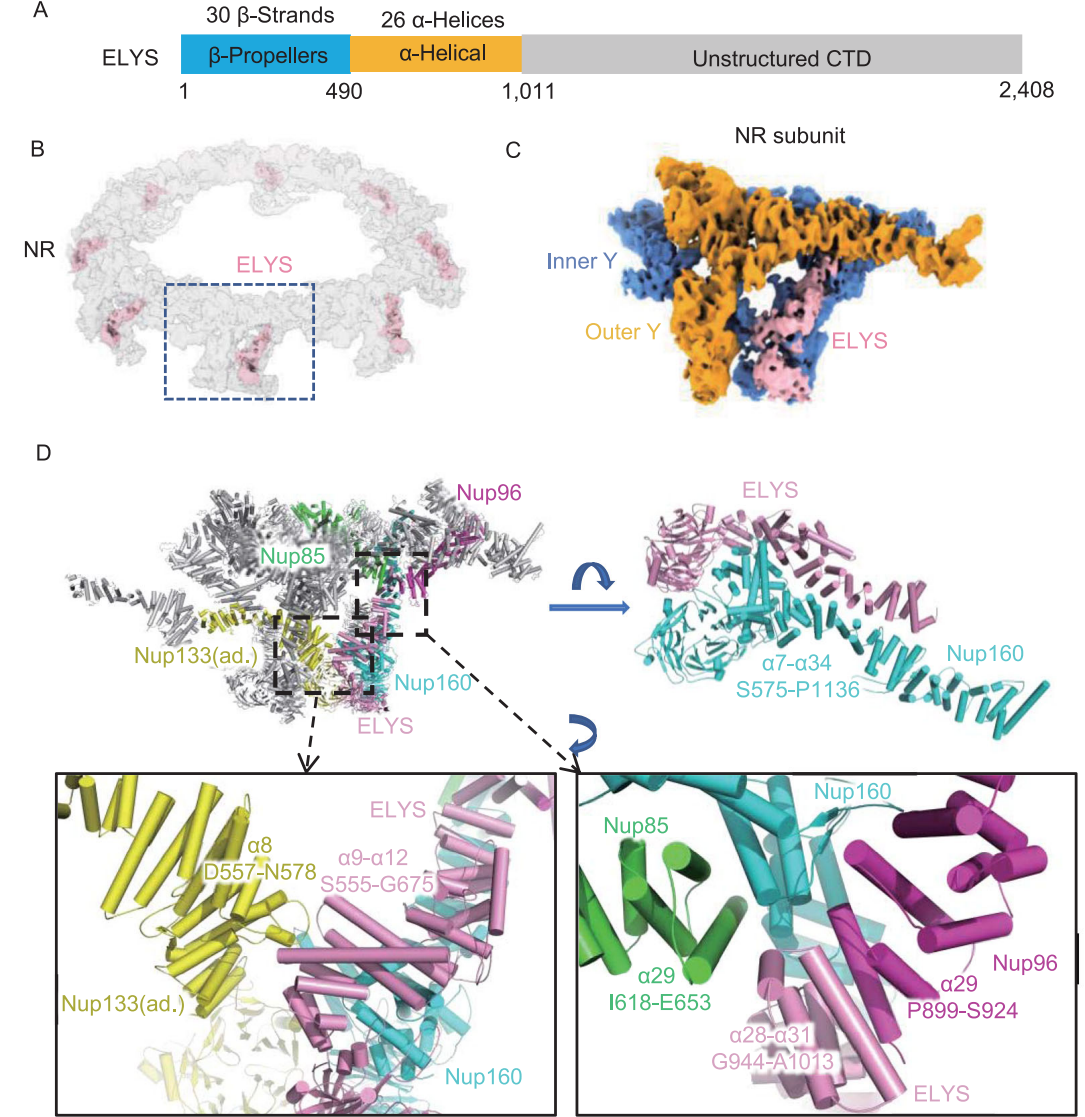
ELYS connects Nup160, Nup96 and Nup85 within the same NR subunit and Nup133 in an adjacent subunit. (A) Domain assignment of *X. laevis* ELYS. (B) Location of ELYS in the NR. (C) Location of ELYS in the NR asymmetric unit. (D) Interaction of ELYS with the convex inner Nup160 and inner Y complex hub in the same NR subunit and the inner Nup133 in the adjacent subunit. All discussed Nups are colored differently. Interaction sites are labeled in detail. Some zoomed-in views are rotated for better display

ELYS in the *X. laevis* NR contains an N-terminal β-propeller domain, a subsequent α-solenoid domain in the NTD and an unstructured domain in the CTD (Fig. 4A). A previous study found that ELYS anchors to the NE through its β-propeller domain and interacts with Nup160 through its α-helical domain; thus, ELYS was thought to be one of the Y complex Nups that localized in the NR (Bilokapic and Schwartz, 2012, 2013; Appen et al., 2015). Stoichiometry research and the cryo-EM structure of the human NPC NR suggested that, in each NR subunit, there are two copies of ELYS binding to both the inner and outer Nup160 (Ori et al., 2013; Appen et al., 2015). However, in this study, according to the high-resolution and isotropic cryo-EM map of the *X. laevis* NR, we found only one copy of the ELYS density situated on the convex side of the inner Nup160 in each NR asymmetric unit (Fig. 4B and 4C). Even when using a mask large enough to cover the possible existing ELYS density on the outer Nup160 during data processing, we still failed to identify the second copy of ELYS in the NR subunit. Therefore, it is possible that ELYS has different copy numbers between humans and *X. laevis*.

The AlphaFold2 predicted model of the ELYS NTD is fitted well into our cryo-EM map of NR (Fig. S6B). It is obvious that ELYS has extensive and tight interactions in both the β-propeller domain and α-solenoid domain with the inner Nup160 (β-propeller domain and α7-α34) (Fig. 4D), suggesting that during the ELYS-mediated NPC assembly process, Nup160 could be easily recruited by ELYS. In addition to the inner Nup160, ELYS also interacts with the inner Nup85, inner Nup96 and inner Nup133 (Fig. 4D). The 9th to 12th α-helices (S555-G675) of ELYS come close to the 8th α-helix (D557-N578) of the inner Nup133 in the adjacent NR subunit, stabilizing the head-to-tail connection of the NR subunits. The C-terminus of the ELYS NTD, α28-α31 helices (G944-A1013), participated in the formation of the inner Y complex hub. In addition to the inner Nup160, this region interacts with the 29th α-helix of the inner Nup85 and the 29th helix of the inner Nup96 (Fig. 4D).

Therefore, our model exhibits the 3D conformations of ELYS in the NR subunit and shows its interactions with four surrounding NR components, i.e., Nup160, Nup133, Nup85 and Nup96. These results show that ELYS significantly contributes to the structural stability of NR and provide clues for understanding the NPC postmitotic assembly process introduced by ELYS.

### Identification of Nup205 in the *X. laevis* CR and NR subunits

**Figure 5:**
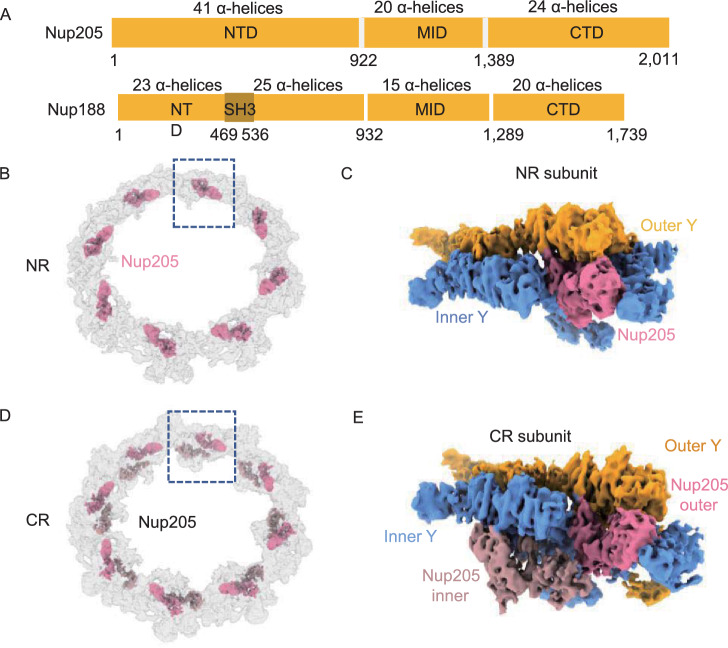
The locations of Nup205 in CR and NR. (A) Domain assignment of Nup205 and Nup188. (B) Location of 8 copies of Nup205 in NR. (C) Location of Nup205 in NR subunit. (D) Location of 16 copies of Nup205 in CR, showing the relative positions of inner and outer Nup205. Nup205 within each subunit is colored differently. (E) Location of 2 copies of Nup205 in the CR subunit

In the cryo-ET map of human NPC (EMD-3103), a question-mark-shaped density was found sandwiched between two Y complexes in both the CR and NR (Appen et al., 2015). This density can be fitted well with the crystal structures of both Nup188 and Nup192 (homolog of Nup205) from *Chaetomium thermophilum* (ctNup188 and ctNup192) (Andersen et al., 2013; Sampathkumar et al., 2013; Stuwe et al., 2014; Stuwe et al., 2015; Lin et al., 2016) because Nup188 and Nup192 share highly similar domain distributions (Fig. 5A). Some studies proposed that this density was more likely to correspond to Nup188, since cross-linking mass spectrometry (XL-MS) revealed cross-linked pairs between Nup85 and Nup188 (Bui et al., 2013; Appen et al., 2015). Other studies suggested that ctNup192 has a long helix (named the tower helix) in its middle domain (MID), which can fit the density well, but the corresponding region in Nup188 lacks structural information (Lin et al., 2016). Most recently, the high-resolution structure of the *X. laevis* NPC attributed the question-mark-shaped densities in the CR to Nup205, mainly according to the density of the tower helix (Huang et al., 2020).

To identify this component and its role in CR and NR more accurately, we first subtracted the densities of both Y complexes from our NR subunit, cropped the only remaining single copy of the question mark-shaped density (Fig. 5B and 5C), and built the model of Nup205 or Nup188 based on the structure predicted by AlphaFold2. In our models, there are 78 helices for Nup188 and 84 helixes for Nup205 (Fig. S11A). Overall, the Nup205 structure can fill this cropped density, and most of its helices fit the map very well, but Nup188 leaves densities unfilled in at least 3 local regions. In the first region at the NTD of Nup205, four α helices (α20, α22, α25 and α26) match well with the local density. However, in this region, Nup188 has an SH3 domain forming several β-strands and could hardly match the map (Andersen et al., 2013) (Fig. S11A). In the second region at the MID of Nup205, the most distinguishable structure of the tower helix (α57), wrapped by two shorter α-helices (α55 and α56), fits well with the map density, but Nup188 has only several short helices in this region that are not long enough to fit (Fig. S11A). In the third region at the CTD of Nup205, two long helices (α79 and α81) with a short helix (α80) that connects them can fill the local map density, but Nup188’s short helices fail to do so (Fig. S11A). Therefore, it can be concluded that the question mark-shaped density represents Nup205 not Nup188 in the NR subunit of *X. laevis* NPCs.

**Figure 6:**
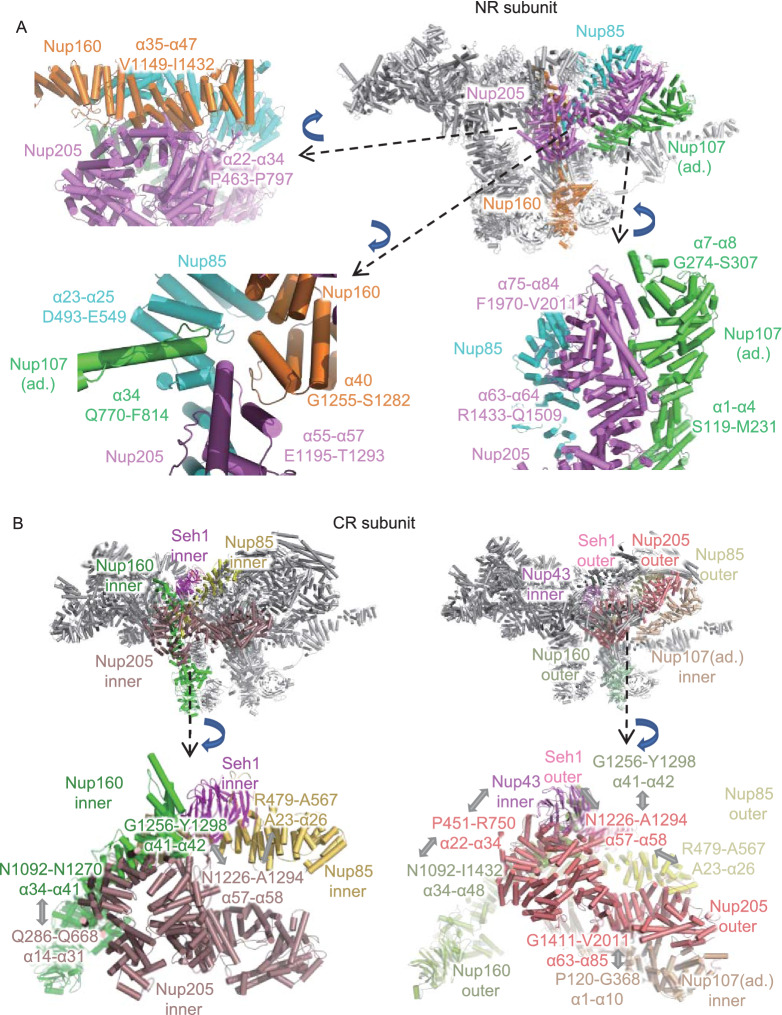
The interaction details of Nup205 in CR and NR. (A) Interaction of Nup205 with surrounding Nups in the same NR subunit and in an adjacent (ad.) subunit. (B) Interaction sites of inner and outer Nup205 to surrounding Nups. All discussed Nups are colored differently. Interaction sites are labeled in detail. All zoomed-in views are rotated for better display

The complete Nup205 model in the NR subunit shows rich interactions with nearby NR components (Fig. 6A). In the NTD, the 22nd to 34th α-helices of Nup205 (P463-P497) connect to the CTD of outer Nup160 (α35 to α47, V1149 to I1432) at the Y complex hub (Fig. 6A). In the MID, the tower helix of Nup205 (α55-α57, E1195-T1293), together with the finger helix of Nup107 (α34, Q770-F814) in the adjacent NR subunit, is inserted into the grooves formed by Nup85 (α23-α25, D493-E549) and Nup160 (α40, G1255-S1282). These four proteins are grouped together in this local region to form a strong interaction network (Fig. 6A). In the CTD, the 63rd and 64th α-helices and 75th to 84th α-helices of Nup205 form tight contacts with the 1st to 4th α-helices and 7th and 8th α-helices of inner Nup107 in the adjacent NR subunit, respectively (Fig. 6A). These results suggest that Nup205 plays an important role in stabilizing the head-to-tail connection of asymmetric units of the NR (Lin et al., 2016).

For the question-mark-shaped density attached to the Y complex arms in CR, similar to the NR subunit, according to the isotropic density map of CR subunit and AlphaFold2 predictions, we modeled and fitted two full-length Nup205 structures into the question mark shaped densities, which are attached to both the inner and outer Y complexes, named the inner and outer Nup205 (Fig. 5D and 5E). As a control, we also tried to model Nup188 according to the same local density. Similar to our above analysis for NR, the structure of Nup205 fits both inner and outer densities much better than Nup188 (Fig. S11B). The most obvious difference is the tower helix in the middle domain of Nup205, which is missing in Nup188. According to the improved model of inner and outer Nup205 in CR, we found that they have quite different interaction features with surrounding Nups. For inner Nup205, the NTD (α14 to α31 helices) contacts inner Nup160 (α41 to α42 helices), while its tower helix region (α57 to α58 helices) interacts with inner Nup85, inner Seh1 and inner Nup160 (Fig. 6B). For outer Nup205, its NTD connects to inner Nup43 and outer Nup160, the tower helix region binds to outer Nup85, outer Seh1 and outer Nup160, and the CTD interacts with Nup107 of the adjacent CR subunit (Fig. 6B). This result showed that inner and outer Nup205 help to stabilize the formation of two Y complexes in a head-to-tail fashion in CR.

### Identification of Nup93 in the CR and NR subunit

**Figure 7:**
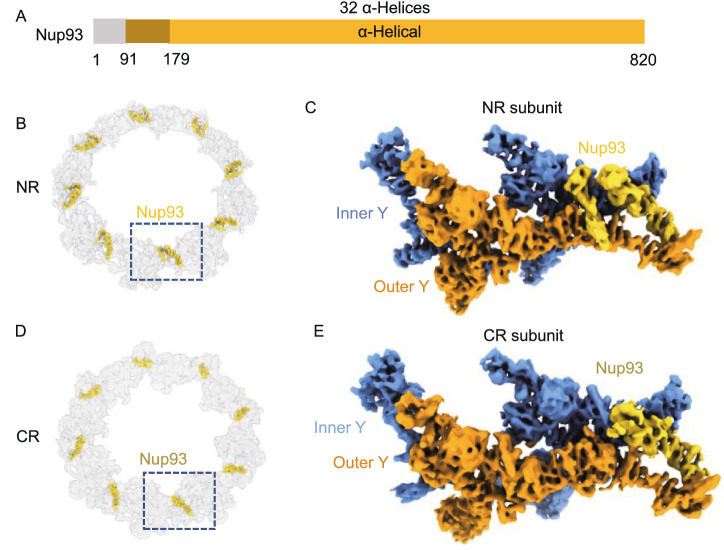
Nup93 acts as a bridge to connect the stems of the inner and outer Y complexes in NR and CR. (A) Domain assignment of the modeled part of Nup93. (B) Location of 8 copies Nup93 in NR. (C) Location of Nup93 in the NR subunit. (D) Location of 8 copies Nup93 in CR. (E) Location of Nup93 in the CR subunit

Nup93 (Nic96 in fungi) (Fig. 7A) has been identified as a building block of the IR complex (IRC) through interactions with channel nucleoporin heterotrimers (CNTs) and Nup205/Nup188 (Stuwe et al., 2015; Lin et al., 2016). Usually, Nup93 is not believed to be localized in the outer rings and involved in CR and NR assembly. However, after assignment of other densities in the NR subunit, we noticed that there was a distinct bridge-like density on the stem region of the two Y complexes (Fig. 7B and 7C). The unknown density at this location has also been depicted in the human NPC (Appen et al., 2015), but there is still no evidence of any Nups being assigned for this density. In our high-resolution NR map, this bridge-like density is divided into two distinct α-helical regions, whose centers are approximately 8 nm apart. The smaller density connects the inner Nup160 and outer Nup107, while the larger density forms an apparent α-solenoid domain to link the inner Nup96 and outer Nup107.

After trials of fitting the predicted structures of all known Nups from *X. laevis* into our cryo-EM map, we found that only Nup93 matched both densities well (Movie S4). The model of Nup93 predicted by AlphaFold2 has two domains, a 5 α-helix NTD and a 32 α-helix CTD, which are connected by a long flexible loop. The special long 5th helix in the Nup93 NTD, which has approximately 50 residues and is even longer than Nup205’s tower helix and Nup107’s finger helix (Boehmer et al., 2008), matches well with the smaller bridge-like density. The cross-correlation (CC) value of the model to map within the mask for Nup93 was calculated to be 0.71 by PHENIX (Liebschner et al., 2019).

**Figure 8:**
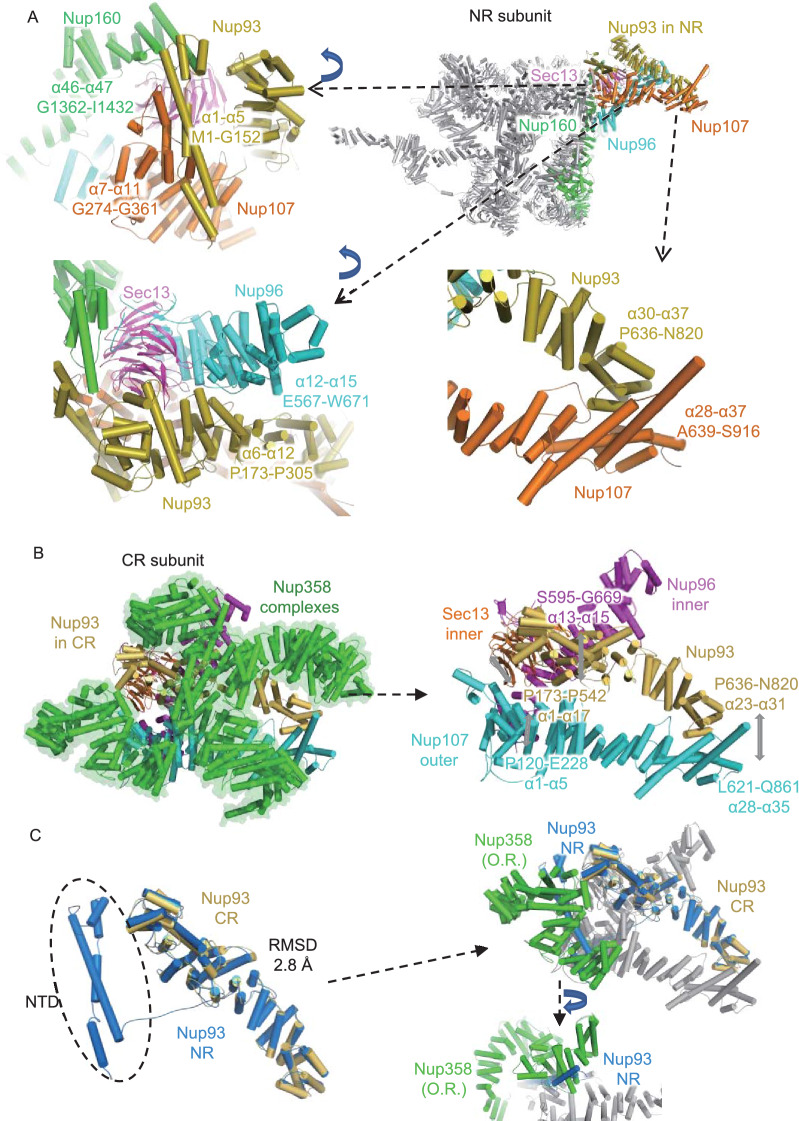
The interaction details of Nup93 in NR and CR. (A) Interaction sites of Nup93 with surrounding Nups in NR. Some zoomed-in views are rotated for better display. (B) Interaction sites of Nup93 with surrounding Nups in CR. (C) Model comparison between Nup93 in CR and NR, showing spatial conflicts between outer right Nup358 and the NTD of Nup93 from NR. All discussed Nups are colored differently. Interaction sites are labeled in detail

The NTD of Nup93 (α1-α5, M1-G152) interacts with the CTD of the inner Nup160 (α46-α47, G1362-I1432) and the outer Nup107 (α7-α11, G274-G361) to connect the stems of the two Y complexes (Fig. 8A). The connecting loop of Nup93 (aa 153–179) allows the separation of the NTD and CTD with a long distance of 8 nm. The Nup93 CTD forms a typical α-solenoid domain, whose 6th to 12th α-helices are in the proximity of the 12th to 15th α-helices of the inner Nup96, and the 30th to 37th α-helices connect to the 28th to 37th α-helices of the outer Nup107. Therefore, both the NTD and CTD of Nup93 play an essential role in connecting the most flexible parts of the two Y complexes in the stem region, thus maintaining the stability of the NR subunit.

Taking a similar surrounding environment into account, there should also be one Nup93 in the CR subunit, yet the corresponding location in the CR subunit has always been regarded as the density for Nup358, so whether there is a similar Nup93 bridge in CR is still unclear. According to the improved cryo-EM map of the CR subunit, we found that when the density corresponding to the five copies of Nup358 was removed from the CR subunit, an unassigned density emerged between the two Y complex stems (Fig. 7D and 7E). Similar to the aforementioned methods, we modeled 31 α-helices of Nup93 at this local density (Fig. S5H). In addition to its interactions with the five Nup358 proteins shown above, it also connects multiple Y complex Nups. In the Nup93 NTD, the α1 to α17 helices (P173 to P542) connect to inner Sec13 and inner Nup96 α13 to α15 helices (S595 to G669). In its CTD, the α23 to α31 helices (P636 to N820) connect to regions near outer Nup107’s finger helix α28 to α35 (L621 to Q861) (Fig. 8B). Therefore, this α-solenoid domain of the Nup93 CR participated in the formation of two Y complex assemblies through its interaction with the stems of both Y complexes in CR.

When comparing the Nup93 models from CR and NR, we found that CR Nup93 has 5 helices missing in the NTD (Fig. 8C), so it seems shorter than NR Nup93. In the superposition of these two models, their main bodies share high structural similarity with an RMSD value of 2.8 Å (Fig. 8C). The location for the missing helices of Nup93 NTD in CR is fully occupied by outer-right Nup358. Therefore, it might be possible that Nup358 binds to the Y complex more tightly than the Nup93 NTD and occupies its interface; then, the density corresponding to the Nup93 NTD is missing in the CR subunit (Fig. 8C).

## Discussion

For a long time, there were two major obstacles in solving the detailed structure of NPCs using the SPA method. One is the anisotropic resolution caused by the preferred orientation problem, and the other is the lack of accurate full-length structures of all Nups in NPC. Here, we combined “side-view” particles and “tilt-view” particles to overcome the insufficient Fourier space sampling problem and used AlphaFold2 to predict all Nups’ structures. Based on the nearly isotropic reconstruction map (Figs. S3 and S4) and the highly accurate predicted models (Figs. S7 and S8), we managed to build the most comprehensive and accurate pseudoatomic model of the NPC outer rings to date. The multiple novel structural features in this model represent a great advance in understanding the assembly of NPCs.

To correctly interpret the map density, the resolution anisotropy must be considered for samples with severe orientational problems, such as the NPC on a flattened NE (Eibauer et al., 2015; Huang et al., 2020). We have made great efforts in solving the NPC structure combined with 0-, 30-, 45-, and 60-degree tilted images, but the 3D-FSC estimates always suggested that the resolutions along the Z axis were only approximately 20 Å, while the resolutions along the other two axes could reach 6~8 Å. This kind of anisotropic map made assignment of the secondary structure features difficult. Thus, to eliminate resolution anisotropy as much as possible, we introduced side-view NPC particles into the dataset and omitted 0-degree tilting images from the very beginning of the image process. This strategy helped to solve this problem by compensating for imperfect Fourier space sampling, improving the Z axis resolution to better than 10 Å (Figs. S3 and S4).

The localization of Nup358 onto both stems of Y complexes was initially established by knockdown experiments of NPC in HeLa cell (Appen et al., 2015). Multiple studies proved that Nup358 is vital for double Y complex arrangement in the CR subunit, since those species lacking Nup358 have only one Y complex left in each CR subunit (Appen et al., 2015; Mosalaganti et al., 2018). For the first time, we found that there should be at least five copies of Nup358 warpped around the stem regions of Y complexes in each CR subunit, and each Nup358 forms extensive but different interactions with surrounding Nups. This result agrees well with previous reports for the importance of Nup358. The reason why NPC needs many copies of Nup358 in the CR region may be related to the large cytoplasmic filaments attached to CR. The high dynamics of cytoplasmic filaments require a stable connection to the CR base, and multiple copies of Nup358 anchoring to the CR could help.

The exportation of mRNAs from the nucleoplasm to the cytoplasm relies on the correct modeling and remodeling of mRNPs, while remodeling of mRNPs on the cytoplasmic side of NPC requires the participation of Gle1, Nup42, Nup214 and DEAD-box helicase (Napetschnig et al., 2009; Port et al., 2015). The mRNP remodeling platform was proposed to project from the cytoplasmic ring onto the nuclear channel to ensure an efficient mRNP remodeling process. Here, we found that there are two Nup214 complexes in each CR subunit of *X. laevis* NPCs, both pointing to the nuclear channel to facilitate correct formation of the mRNP export platform. However, in yeast, the bases of this mRNP remodeling platform were reported to be a Nup82 holo-complex or a P-shaped architecture (Fernandez-Martinez et al., 2016; Allegretti et al., 2020). The structural difference of the Nup214 complex in different species may be related to the different copy numbers of Y complexes in CR. In yeast, NPC has only one Y complex in each CR subunit, so it cannot support the rake-shaped structure of the Nup214 (Nup159) complex found in this study, since parallel formation of Nup214 complexes requires the connection to both inner and outer Nup85 simultaneously. Thus, in yeast, Nup159 needs to form a different confirmation, such as the holo-complex dimer, to anchor onto the CR region. Moreover, the two copies of Nup214 complexes in parallel seem to provide a much denser arrangement of FG-repeat domains inside the nuclear channel, which would lead to a more efficient mRNP exporting process in vertebrates.

In contrast to vertebrate NPCs, NPCs in fungi exhibit less conservation in the overall shape of their outer rings. In each asymmetric unit of the NPC from *C. reinharadtii* or *S. pombe*, the CR has only one Y complex, and the NR has two Y complexes in each asymmetric unit, while in *S. cerevisiae*, both outer rings have only one Y complex in each asymmetric unit (Appen et al., 2015; Mosalaganti et al., 2018; Allegretti et al., 2020). Nup358 is located in the stem region of the CR, similar to Nup93 in the NR, and knockdown of Nup358 could cause a lack of the formation of two Y complexes in the NPC CR (Appen et al., 2015; Mosalaganti et al., 2018). This proved that Nup358 is necessary to maintain the two Y complex conformations in the CR. However, the role of Nup93 in the NR might be different from that of Nup358 in the CR. Among the NR maps in different species (Fig. S12), we found that the Nup93 corresponding density was present only at higher organisms, such as vertebrates, and was missing in *S. pombe* and *C. reinharadtii*, the NPCs of which also had two Y complexes in each NR asymmetric unit (Appen et al., 2015; Mosalaganti et al., 2018). Therefore, Nup93 does not seem necessary to maintain the two Y complex conformations in the NR.

In all species of humans, toads, algae, and yeast (*S. pombe*), ELYS is present in the genome as a full-length protein, or in a truncated form, or as a homolog protein ELY5 (Bilokapic and Schwartz, 2012, 2013; Appen et al., 2015; Mosalaganti et al., 2018). ELYS has been proven to participate in Y complex hub formation and play a role in connecting adjacent Y complexes. In yeast, ELY5 has only α-helical regions and thus makes no contribution to anchoring the Y complex onto the NE but may play a similar role in stabilizing the Y complex hub. According to sequence alignment, ELY5 contains α-helical regions corresponding to the 13th to 30th α-helices of ELYS, which cover the major interaction sites of the ELYS α-solenoid domain with respect to Nup160. ELYS is highly evolutionarily conserved in both structures and functions, indicating that the existence of ELYS in NPCs is essential for NPC assembly and the evolution of the NR from single-cell organisms to vertebrates.

According to our improved models of CR and NR, there are two copies of Nup205 in the CR subunit and one copy in the NR subunit. In the reported model of IR, there should be 4 Nup188 or Nup205 in each IR subunit (Lin et al., 2016; Kosinski et al., 2016). Meanwhile, according to stoichiometry data for Nups reported previously, the total amount of Nup205 in NPC is roughly twice of Nup188 (Ori et al., 2013). Hence, it is impossible that there is only Nup205 or only Nup188 in IR, and it should be the combination of Nup205 and Nup188, but the exact result requires a high-resolution model of IR in the future.

In summary, we solved the cryo-EM map of the *X. laevis* NPC CR and NR at an isotropic resolution of approximately 8 Å and obtained a more accurate and complete model at the secondary structure level. The revealed new structural details advanced our understanding of the detailed organization and assembly of vertebrate NPCs.

## Materials and methods

### Sample preparation

Ovaries were removed from narcotized mature female *X. laevis*, stage VI oocytes were isolated, and NE was applied onto the grid in ice-cold HEPES buffer (83 mmol/L KCl, 17 mmol/L NaCl, 10 mmol/L HEPES, pH 7.5). Before plunge freezing, the sample on the grid was cross-linked with 0.15% glutaraldehyde for 10 min on ice. After the cross-linking process, the grid was blotted and vitrified by plunge freezing into liquid ethane by Vitrobot Mark IV (Thermo Fisher Scientific, USA) at 100% humidity, and all grids were stored in liquid nitrogen before imaging.

The animal experiments were performed in the Laboratory Animal Center of Peking University in accordance with the National Institutes of Health Guide for the Care and Use of Laboratory Animals and according to guidelines approved by the Institutional Animal Care and Use Committee at Peking University.

### Cryo-EM data acquisition

After screening in a Talos Arctica 200 kV transmission electron microscope (TEM) (Thermo Fisher Scientific, USA), the good grids were mounted into a Titan Krios 300 kV TEM (Thermo Fisher Scientific, USA) for imaging. A total of 8,745 images were collected at a nominal magnification of 64,000×, resulting in a calibrated physical pixel size at the specimen level of 2.24 Å. For images at a tilting angle at 0/30/45/60 degrees, the total dose was set to be 100 or 120/60/80/100 e-/Å2; the movies were recorded on a 0.5 s per frame base, and the exposure time of these collected datasets was set to be 28.5 or 34.5/21.5/41 or 28.5/36 or 35 s (Table S1). All movies were recorded by a Gatan K2 Summit Direct electron detector (DDD) (Gatan Company, USA) under super-resolution mode equipped with a post column GIF Quantum filter, whose slit width was set to be 20 eV. SerialEM with in-house scripts was used for data collection with the defocus value set between 1.0 μm and 4.0 μm (Mastronarde, 2005; Wu et al., 2019).

### SPA image processing

Motion correction and dose weighting were performed by MotionCor2 (Zheng et al., 2017). Particle picking was done by using RELION ver3.0 prior to CTF estimation (Zivanov et al., 2018). Only particles with apparent features such as NPC were kept for further processing. CTF estimation was performed using Gctf, goCTF, or Warp on a per-particle basis (Zhang, 2016; Su, 2019; Tegunov and Cramer, 2019).

The image alignment processing of CR and NR were basically the same, both using RELION 3.0 unless stated specifically (Zivanov et al., 2018). Taking CR as an example, prior to alignment, we docked the previously reported model of the CR from human NPC (Protein Data Bank entry PDB 5A9Q) into a full NPC map obtained using a similar approach described previously (Huang et al., 2020), and segmented the surrounding density using Chimera, the segmented part was used to generate a mask solely covering the CR part in our map (Pettersen et al., 2004; Appen et al., 2015). Then, refinement of the CR on the binned 4 level using this CR mask was performed. The initial reference was generated by low pass filtering the reported human NPC structure to 60 Å, and C8 symmetry was applied during refinement. The refinement of CR on the binned 4 level reached a final resolution of 29 Å. Then, using refined orientations and shifts, CR particles at the binned 2 level were extracted with a box size of 400 pixels. After extraction, reconstruction was performed for the extracted particles to generate a mask solely covering the CR region. Using a similar strategy as at the binned 4 level of CR particles, refinement was performed and reached a final resolution of 23 Å.

Then, the alignment was performed at the subunit level, as no significant gain of resolution would be achieved for the whole CR by decreasing the binning levels. The relative coordinate of the CR subunit to the CR box center was determined using Chimera, and then we used a modified version of a block-based reconstruction script (Script S1) to generate a RELION star file containing orientations and updated defocus values of each subunit (Pettersen et al., 2004; Zhu et al., 2018). Then, we extracted the subunit particles and first ran a reconstruction job to ensure everything was correct. The model of PDB 5A9Q was used to generate a mask solely covering regions corresponding to one asymmetric unit, and refinement using this mask was performed to reach a resolution of 10.7 Å for the CR subunit. Extraction of binned 1 particle was performed using refined shifts and orientations from refinement of binned 2 particles, with a box size of 320 pixels. Similar to what was done for binned 2 CR subunit particles, first, a reconstruction was performed to obtain an initial reference, and a mask solely covering one subunit was generated. Refinement at this stage reached a resolution of 9.8 Å. Next, we ran Bayesian polishing of all particles. The output star file was separated into multiple files, each containing particles corresponding to individual stage tilting angles. Then, 3D classification was performed for these individual tilts, using the refined map as a reference. After classification, all particles corresponding to the best class in different jobs were selected and merged, and then an auto-refinement was performed for the classified particles and reached a resolution of 8.8 Å. Then the output star file and corresponding map were subjected to CryoSPARC for final refinement using its local refinement tool, resulting in a final resolution of 8.7 Å (Punjani et al., 2017). Similarly, a mask covering the most rigid part of the CR subunit was created, subjected to CryoSPARC for local refinement using the same particle dataset and reached a final resolution of 8 Å for the CR core region (Punjani et al., 2017). A similar strategy was applied to the CR Nup358 region and reached a final resolution of 8.9 Å. The validation of the map and model quality of this research was performed using 3D-FSC and Phenix (Afonine et al., 2012; Tan et al., 2017) (Fig. S1).

For the NR subunit, after the last round of refinement in RELION, the output particles, map, and mask were transferred into CryoSPARC to perform local refinement, which reached a final resolution of 8.1 Å (Punjani et al., 2017; Zivanov et al., 2018). A rather stable NR core region was identified by investigating the local resolution distribution of this 8.1 Å map using RELION-3.0, and a mask covering only this region was created (Zivanov et al., 2018). Then, a local refinement of the NR core region was performed using the same data as the 8.1 Å map, which resulted in a final resolution of 7.8 Å (Punjani et al., 2017). A similar strategy was applied to the NR Nup133 region of the NR and resulted in a resolution of 8.6 Å (Fig. S2).

### Modeling of NPC CR and NR

The full version of AlphaFold2 was installed as instructed with all databases downloaded (Jumper et al., 2021). All the structures of NPC CR and NR Nups from *X. laevis* or *X. tropicalis* (Nup160 and Nup96) (Fig. S13), were predicted by AlphaFold2 using the recommended parameters. Briefly, the value of Max_template_hits was set to 20, Relax_energy_tolerance was set to 2.39, Relax_stiffness was set to 10, and Relax_max_outer_iterations was set to 20. For each Nup, a total of 5 relaxed structures were predicted, and the prediction with the highest confidence was selected as the starting model for the next refinement.

Then we performed stepwise MDFF simulations to refine each Nup model according to the corresponding local density in the CR subunit. A timestep of 1 fs was used throughout the simulation. Langevin dynamics were adopted at a temperature of 310 K. The equilibration step for energy minimization was performed on the initial model for 1000 steps before the refinement run. The refinement runs were performed for 3,000 ps, which corresponds to 3,000,000 simulation steps, and the gridForceScale values were gradually increased from 0.3 to 0.7 during the refinement. All simulations were performed using CHARMM36m forcefields (Huang et al., 2017). Electrostatic calculations were treated with particle mesh Ewald (PME). A cutoff of 12 Å was chosen for short-range van der Waals interactions. NAMD (Phillips et al., 2005) was used as the MD engine throughout all simulations.

All the CR and NR components were assembled in COOT (Emsley et al., 2010) for manual adjustment according to the overall density map (Fig. S7 and S8). Then, the whole model of the CR or NR subunit was refined using PHENIX.real_space_refine (Liebschner et al., 2019). Data collection statistics and refinement statistics are given in Table S1. All figures in this study were generated by PyMol, Chimera and ChimeraX (Pettersen et al., 2004; Goddard et al., 2018).

## Supplementary Information

Below is the link to the electronic supplementary material.

Movie S1 (MP4 7704 KB)

Movie S2 (MP4 21444 KB)

Movie S3 (MP4 35062 KB)

Movie S4 (MP4 2279 KB)

Supplementary figures and tables (PDF 28774 KB)

Script S1 (PY 7 KB)

## Data Availability

The Electron Microscopy Database (EMD) accession codes of the CR subunit region, CR core region and CR Nup358 region are EMD-32056, EMD-32060, EMD-32061, respectively. The Protein Data Bank (PDB) accession code of the model of the CR subunit is 7VOP. The EMD accession codes of the NR subunit region, NR core region and NR Nup133 region are EMD-31891, EMD-31892, EMD-31893, respectively. The PDB accession code of the model of the NR subunit is 7VCI.

## Abbreviations

CC: cross-correlation
CNTs: channel nucleoporin heterotrimers
*CR*: cytoplasmic ring
*C. reinharadtii*: *Chlamydomonas reinharadtii*
cryo-EM: cryo-electron microscopy
cryo-ET: cryo-electron tomography
CTD: c-terminal domain
DDD: direct electron detector
DIM: domain invasion motifs
EMD: the Electron Microscopy Data Bank
FSC: Fourier shell correlation
*H. sapiens*: *Homo sapiens*
INM: inner nucleus membrane
IR: inner ring
IRC: IR complex
MDFF: molecular dynamics flexible fitting
MID: middle domain
mRNP: messenger ribonucleoprotein
NE: nuclear envelope
NPC: nuclear pore complex
NR: nuclear ring
NTD: N-terminal domain
Nup: nucleoporin
ONM: outer nucleus membrane
PDB: Protein Data Bank
PME: particle mesh Ewald
RMSD: root-mean-square error
*S. cerevisiae*: *Saccharomyces cerevisiae*
SPA: single particle analysis
*S. pombe*: *Schizosaccharomyces pombe*
STA: subtomogram averaging
3D-FSC: three-dimensional Fourier shell correlation
3HB: three helices bundle
TEM: transmission electron microscope
*X. laevis*: *Xenopus laevis*
XL-MS: cross-linking mass spectrometry
*X. tropicalis*: *Xenopus tropicalis*

## Code availability

Modified version of block-based reconstruction script is available upon supplementary files (Script S1).
