# Supplementary material for: 8 Å structure of the outer rings of the Xenopus laevis nuclear pore complex obtained by cryo-EM and AI
Source: Protein Cell. 2022 Jan 11;13(10):760–77. doi: 10.1007/s13238-021-00895-y (PMC9233733; doi:10.1007/s13238-021-00895-y)
Supplement: Supplementary file 5 — Supplementary figures and tables (PDF 28774 KB) [file 13238_2021_895_MOESM5_ESM.pdf]

Supplementary figures and tables

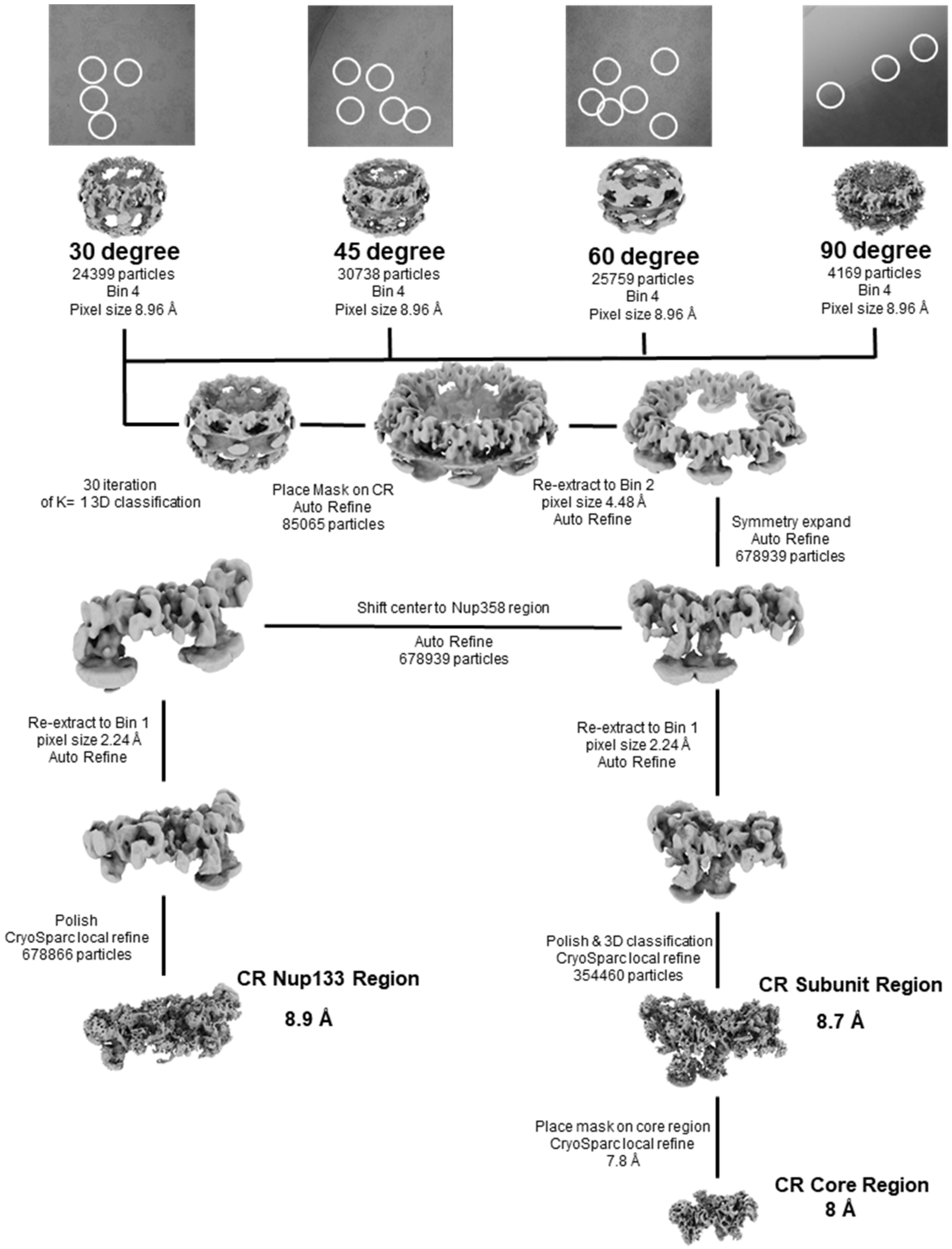

Figure S1. Data processing workflow of the CR from the *X. laevis* oocyte NPC.

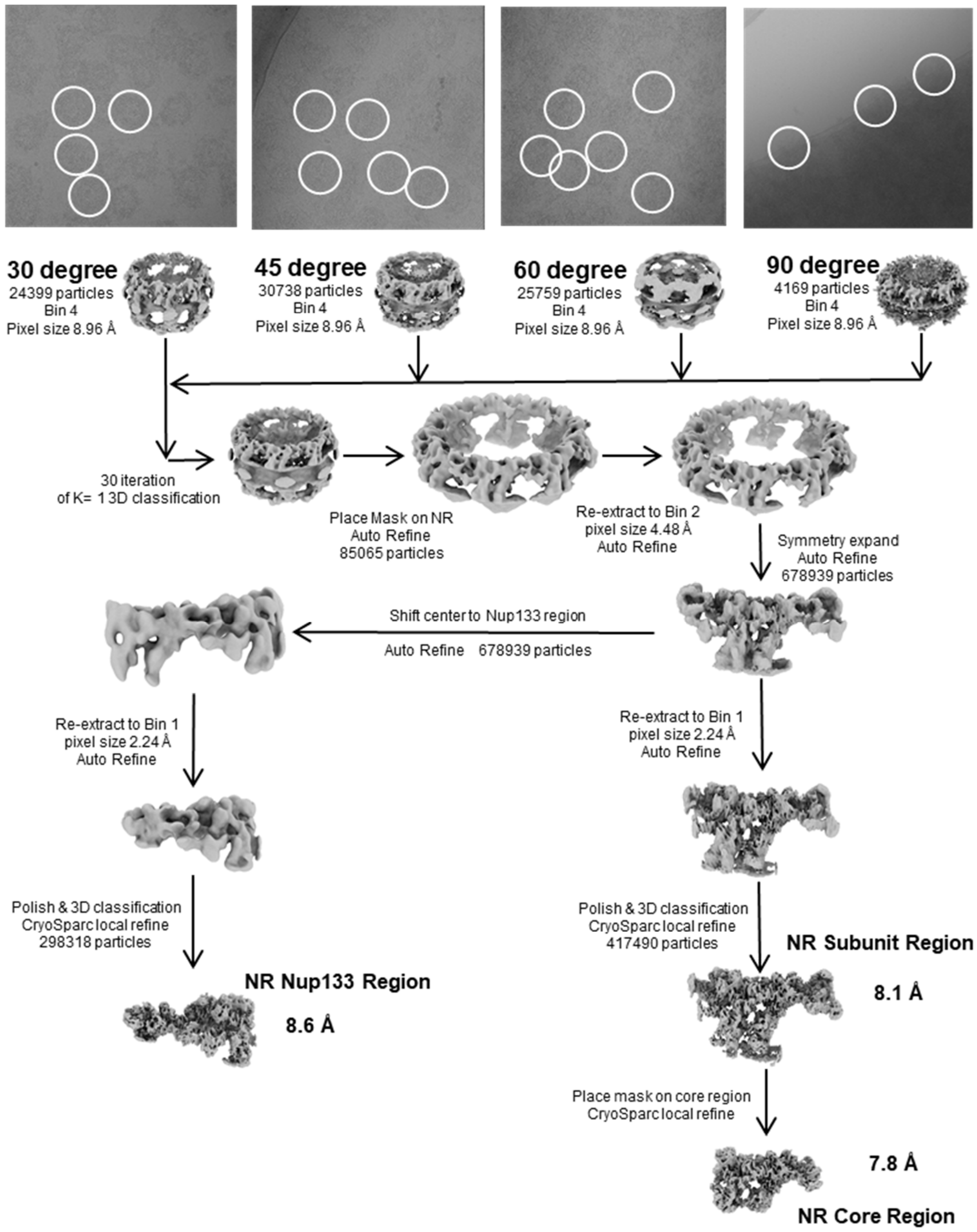

Figure S2. Data processing workflow of the NR from the *X. laevis* oocyte NPC.

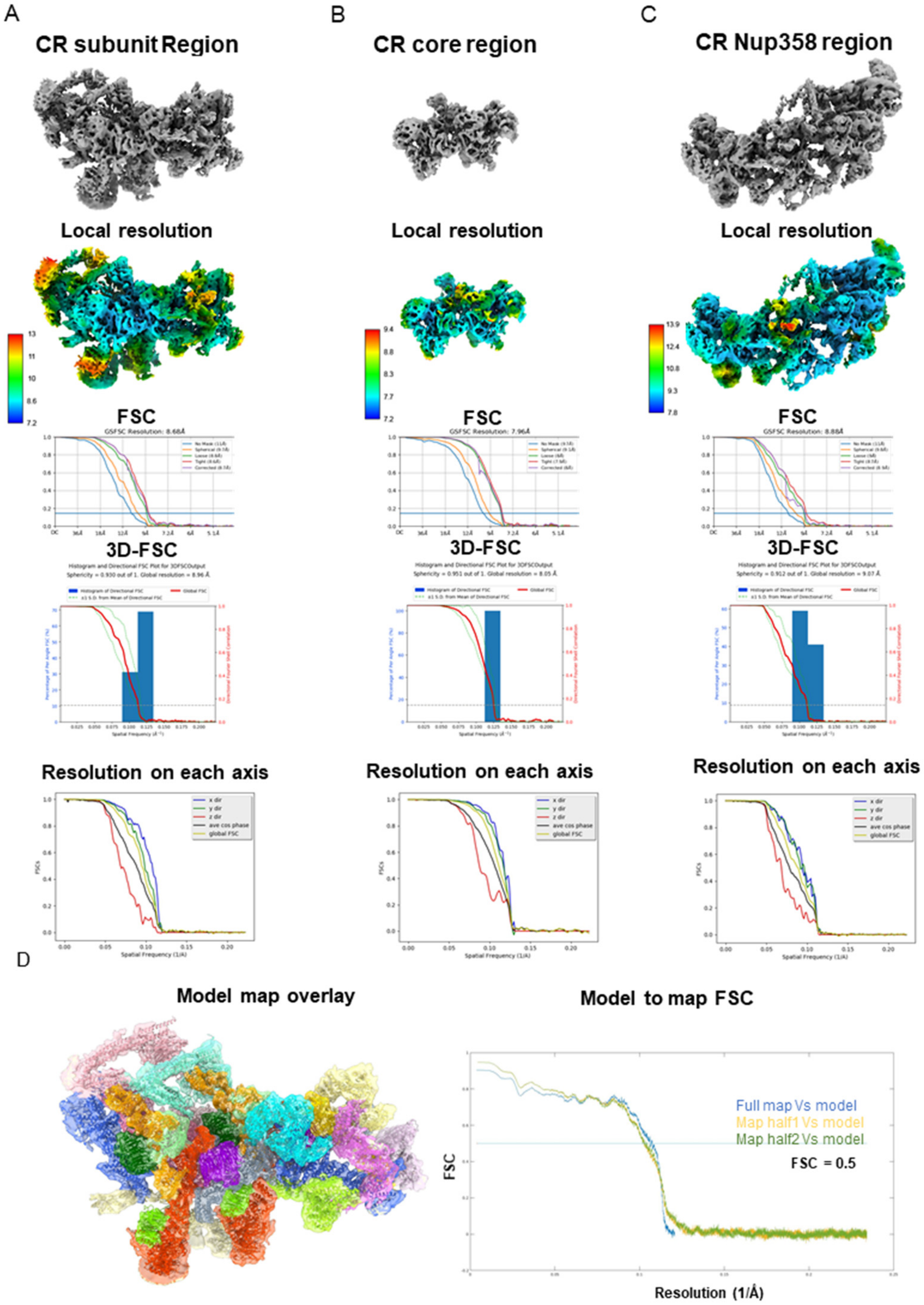

Figure S3. Assessments of cryo-EM maps of the CR subunit. (**A, B, C**) Map display, local resolution estimation, FSC, 3D-FSC and directional FSC of the CR subunit region, CR core region and CR Nup358 region. (**D**) Map model overlay of the CR subunit, and map versus model FSC estimation.

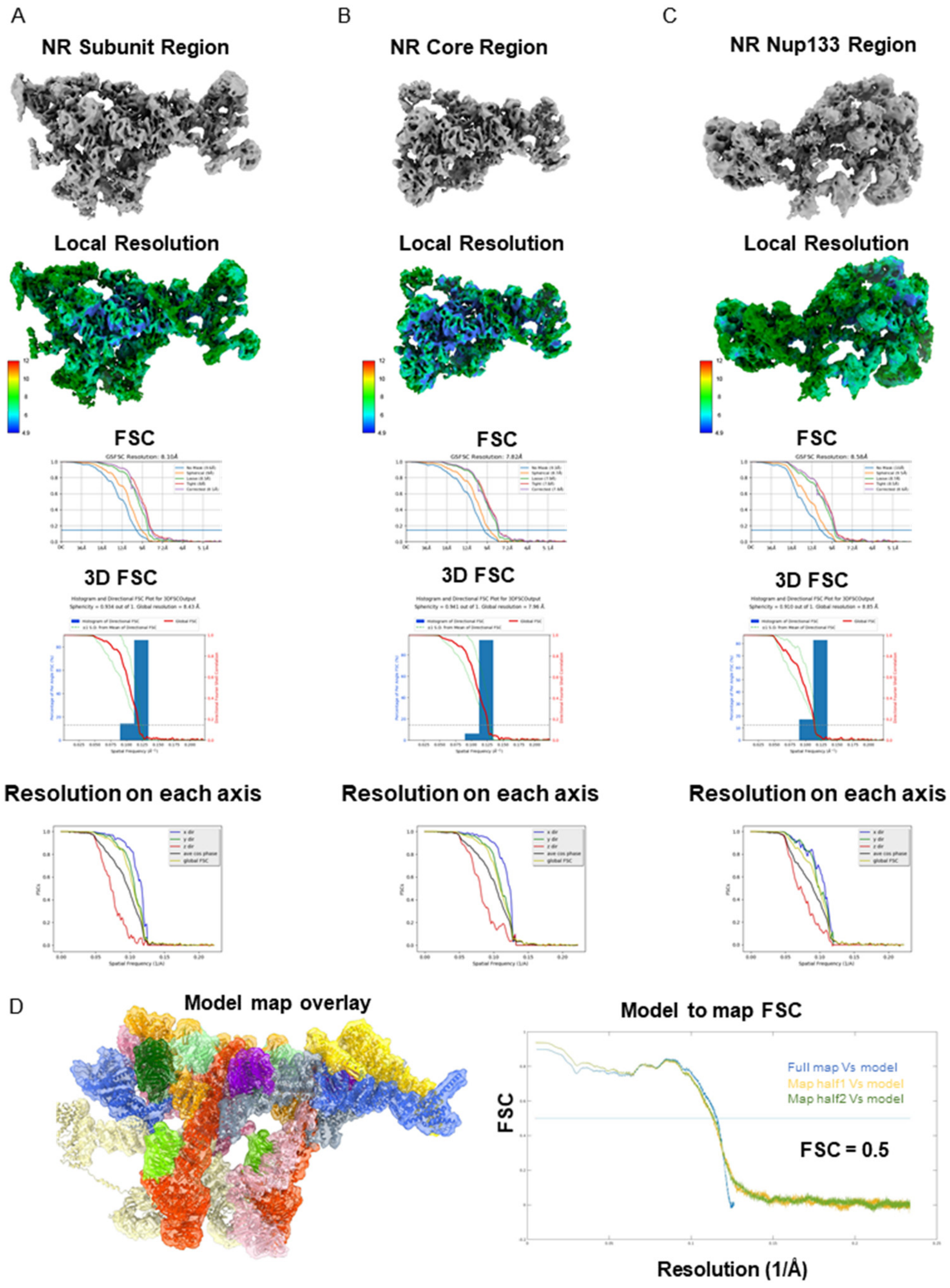

Figure S4. Assessments of cryo-EM maps of the NR subunit. (**A, B, C**) Map display, local resolution estimation, FSC, 3D-FSC and directional FSC of the NR subunit region, NR core region and NR Nup133 region. (**D**) Map model overlay of the NR asymmetric unit, and map versus model FSC estimation.

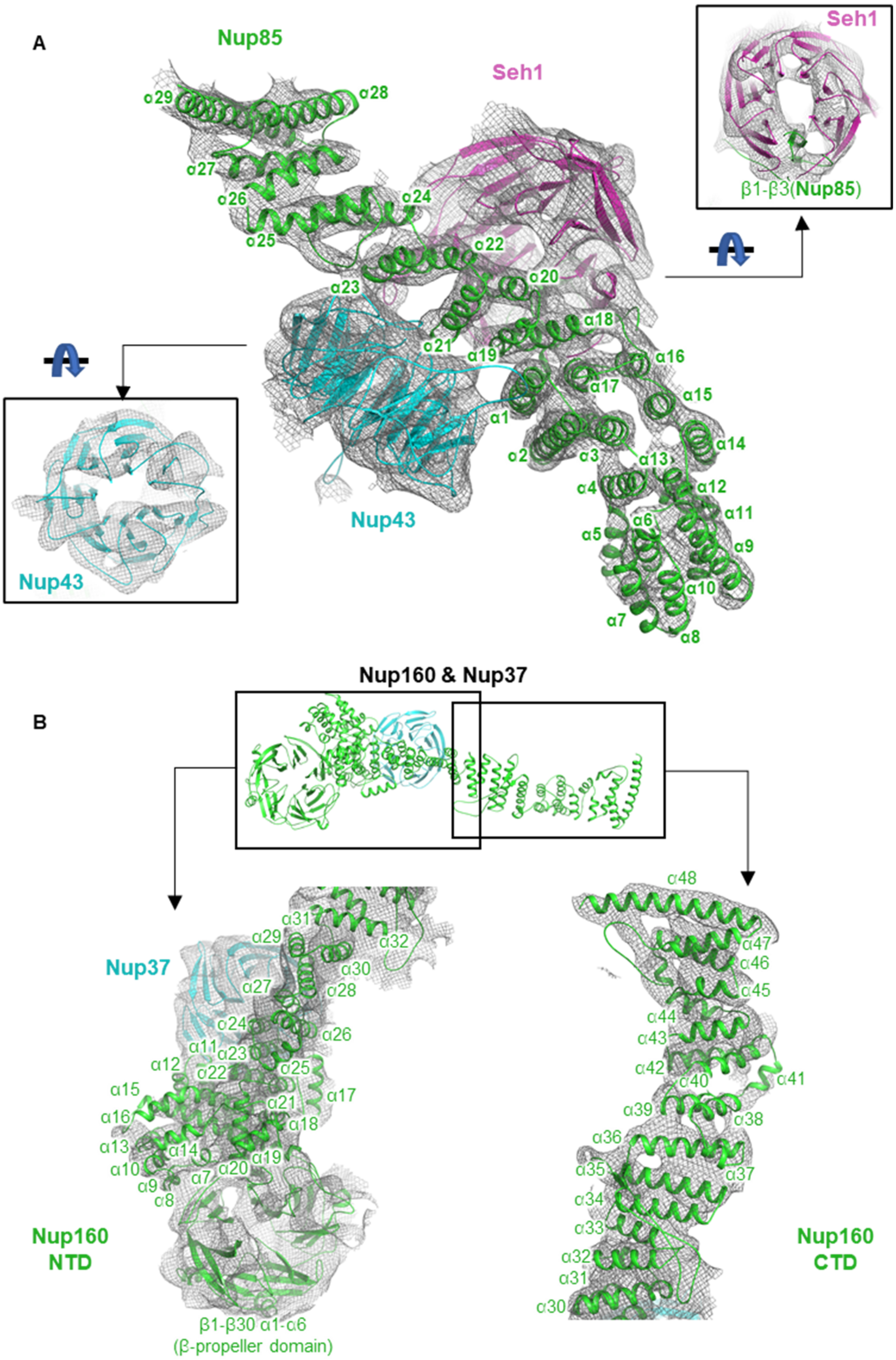
A
Nup85
Seh1
Seh1
β1-β3(Nup85)
Nup43
Nup43
B
Nup160 & Nup37
Nup37
Nup160
NTD
β1-β30 α1-α6
(β-propeller domain)
Nup160
CTD

C

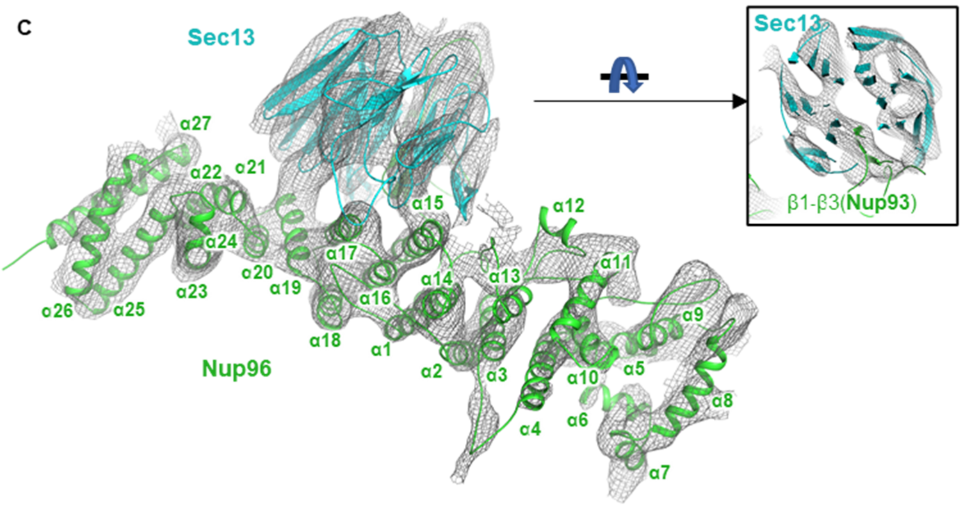
Sec13
Sec13
β1-β3(Nup93)
α27
α22
α21
α24
α15
α12
α17
α20
α19
α23
α14
α13
α11
α16
α26
α25
α18
α1
α9
Nup96
α2
α3
α10
α5
α8
α4
α6
α7

D

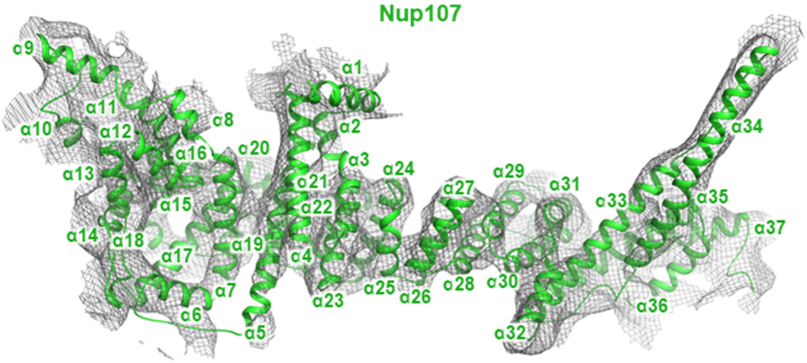
Nup107
α9
α1
α11
α10
α12
α8
α2
α16
α20
α3
α13
α24
α34
α21
α29
α31
α27
α15
α22
α33
α35
α37
α14
α18
α19
α17
α4
α7
α23
α25
α26
α28
α30
α36
α6
α5
α32

E

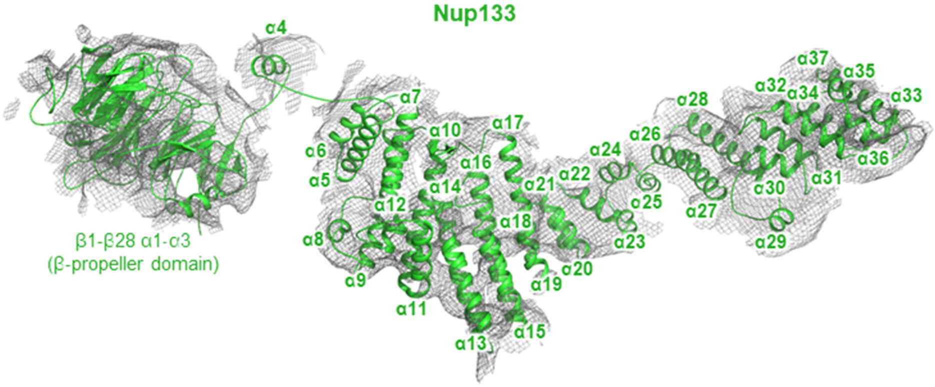
Nup133
α4
α37
α35
α32
α34
α33
α7
α28
α10
α17
α6
α26
α24
α16
α36
α5
α22
α21
α31
α12
α14
α30
α25
α27
α18
β1-β28 α1-α3
(β-propeller domain)
α8
α23
α29
α20
α9
α19
α11
α13
α15

F

Inner Nup205

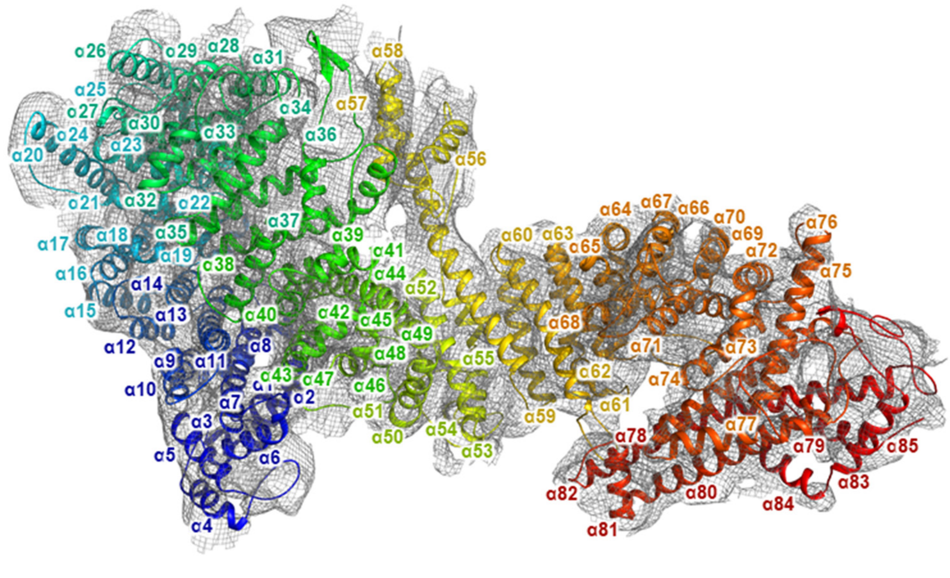

G

Outer Nup205

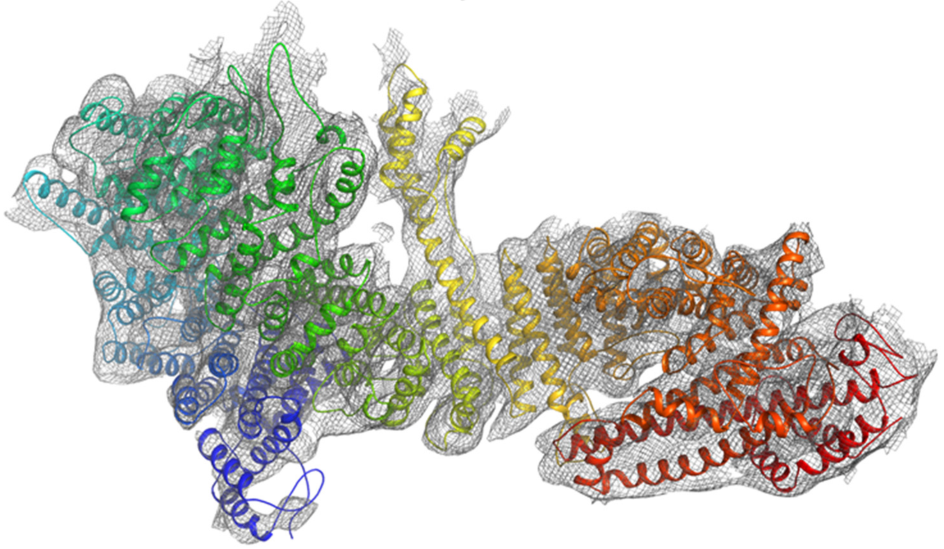

H

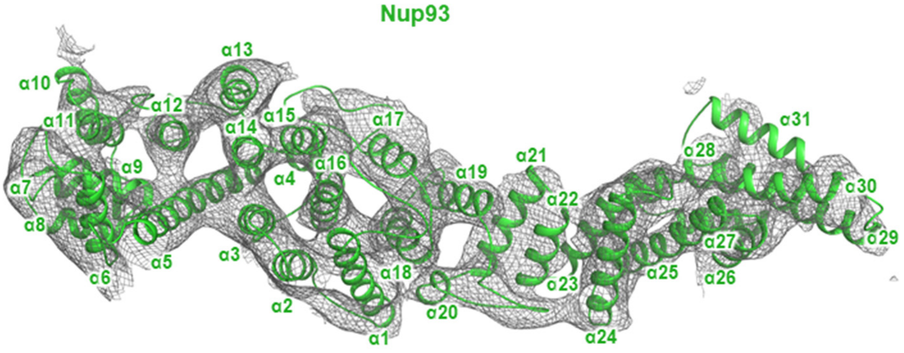

Nup93
α13
α10
α12
α11
α14
α15
α17
α9
α4
α16
α19
α21
α28
α31
α7
α22
α30
α8
α29
α27
α5
α3
α18
α25
α26
α6
α23
α2
α20
α24
α1

I

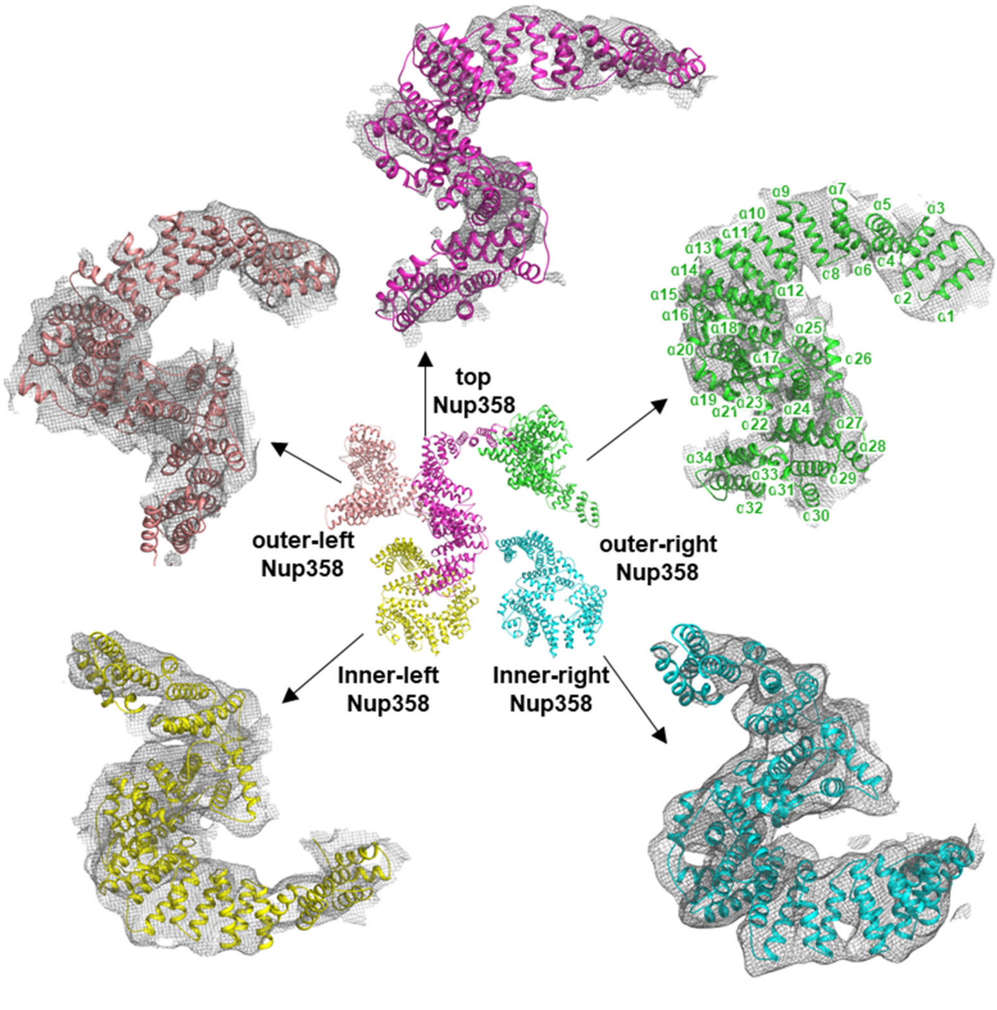

top
Nup358
outer-left
Nup358
outer-right
Nup358
Inner-left
Nup358
Inner-right
Nup358
α9
α7
α10
α5
α3
α11
α13
α8
α6
α4
α14
α12
α15
α2
α16
α1
α18
α25
α20
α17
α26
α19
α21
α23
α24
α22
α27
α28
α34
α33
α31
α29
α32
α30

J

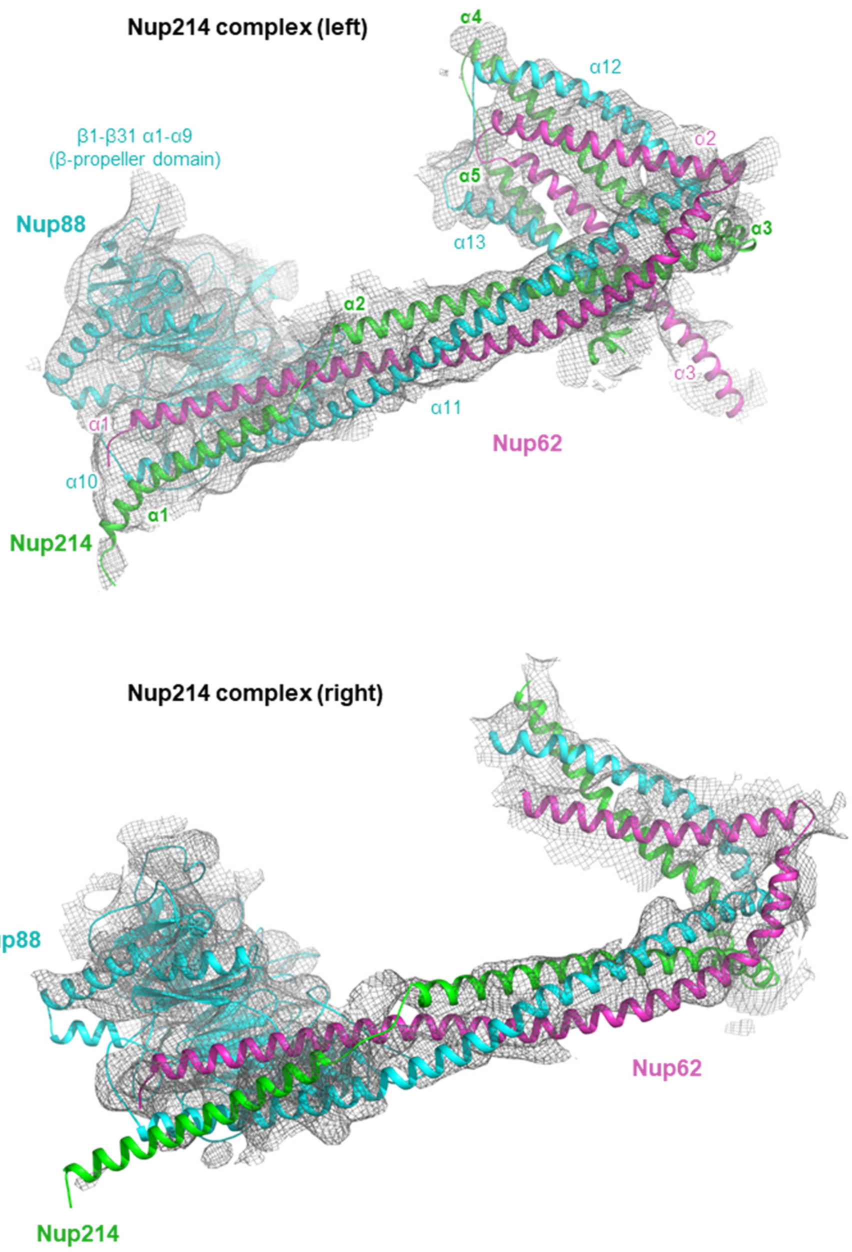

Figure S5. Model building quality and secondary structure assignment of the CR subunit. (A-J) Model building quality of Nup85, Seh1, Nup43, Nup160, Nup37, Sec13, Nup96, Nup107, Nup133, Nup205, Nup93, Nup358 and Nup214 complexes.

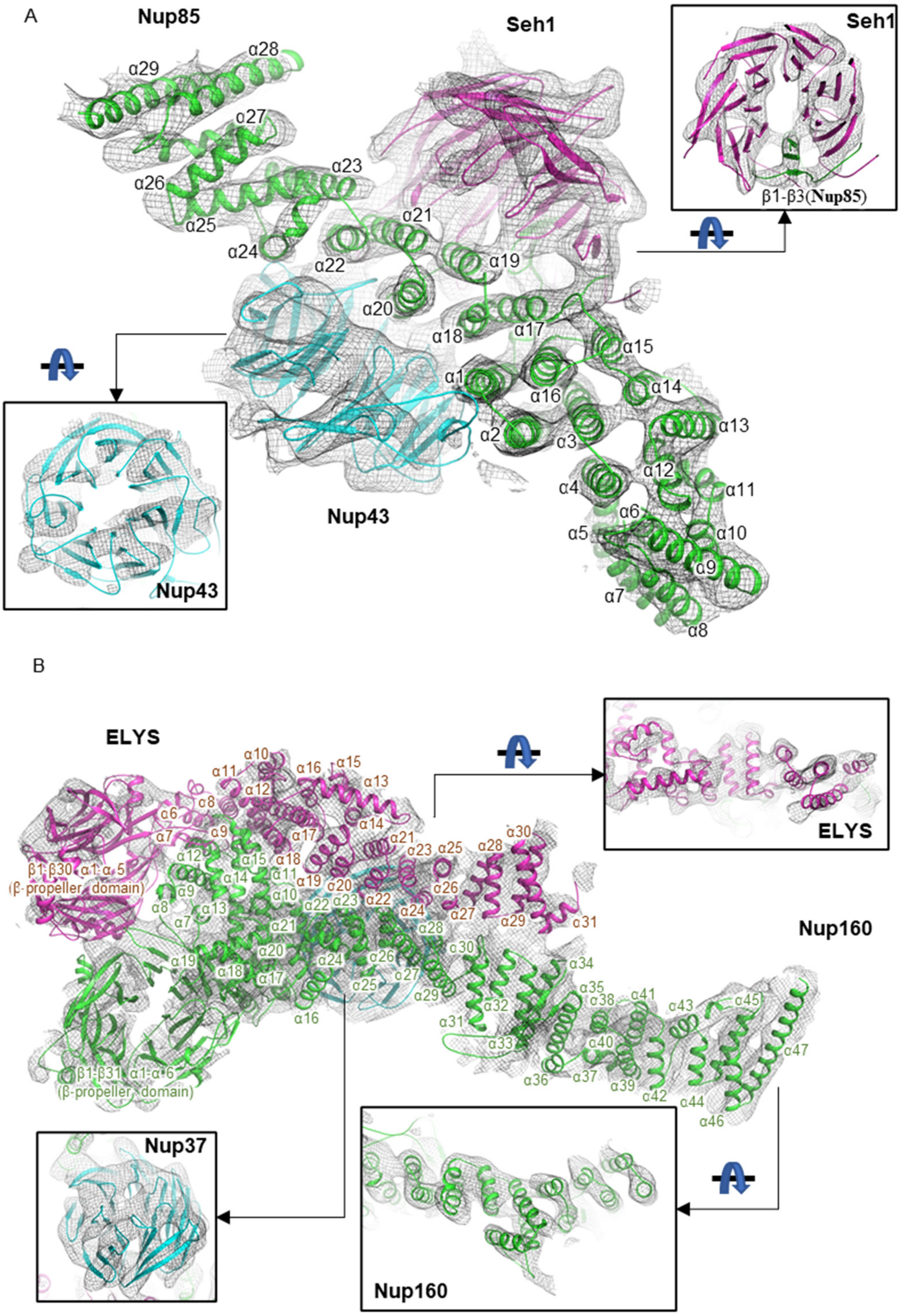
A
Nup85
Seh1
Seh1
β1-β3(Nup85)
Nup43
Nup43
B
ELYS
ELYS
β1-β30 α1-α5
(β-propeller domain)
β1-β31 α1-α6
(β-propeller domain)
Nup160
Nup37
Nup160

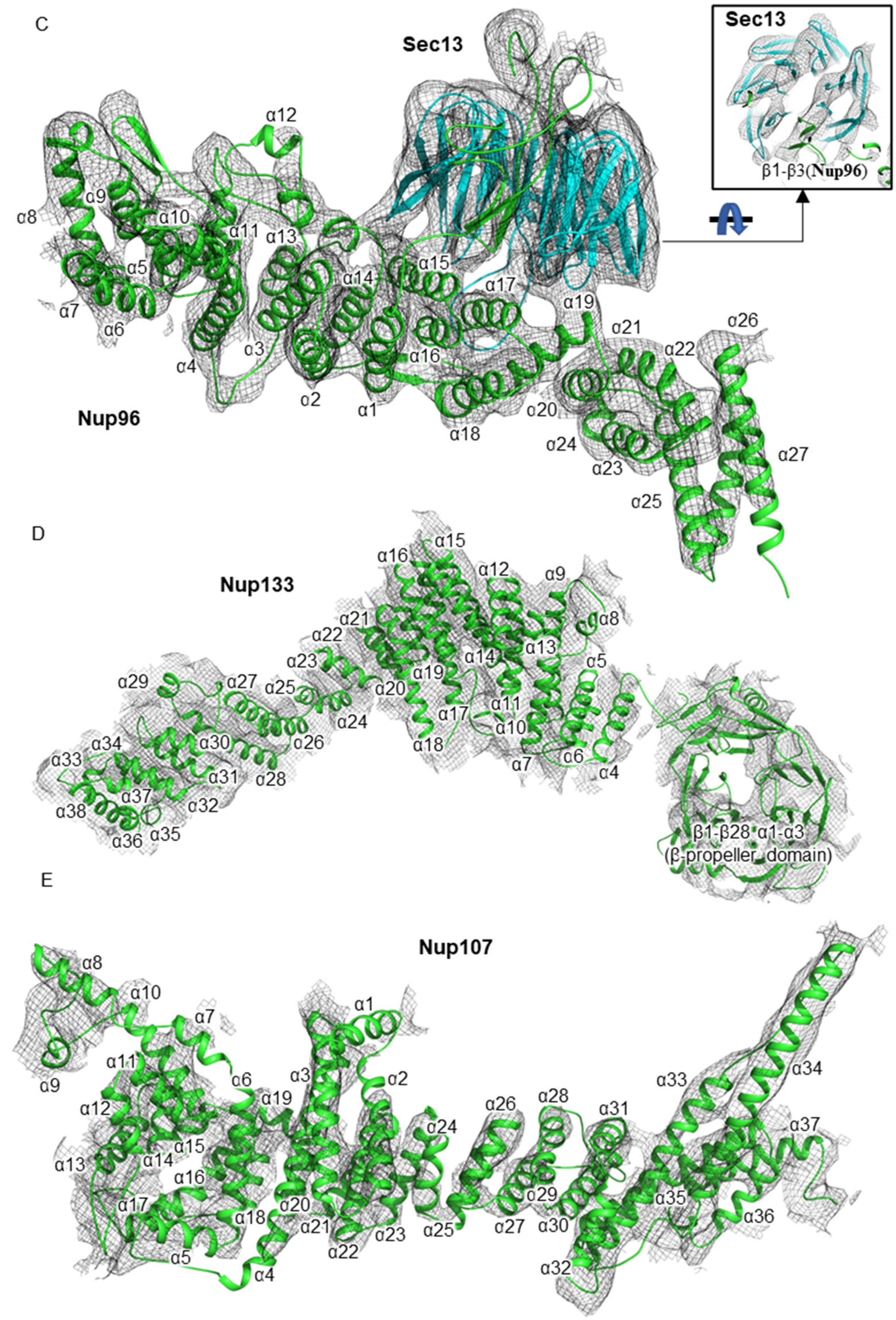
C
Sec13
Sec13
β1-β3(Nup96)
α12
α8
α9
α10
α11
α13
α5
α14
α15
α17
α19
α7
α6
α21
α26
α22
α4
α3
α16
α2
α1
α20
α18
Nup96
α24
α27
α23
α25
D
Nup133
α16
α15
α12
α9
α8
α22
α21
α13
α23
α14
α5
α19
α29
α27
α25
α20
α24
α11
α17
α10
α34
α30
α26
α18
α7
α6
α4
α33
α31
α28
α37
α32
α38
α36
α35
β1-β28 α1-α3
(β-propeller domain)
E
Nup107
α8
α10
α7
α1
α9
α11
α6
α3
α2
α19
α33
α34
α12
α26
α28
α31
α24
α37
α15
α13
α14
α16
α29
α35
α17
α18
α20
α21
α23
α25
α27
α30
α36
α22
α5
α4
α32

F

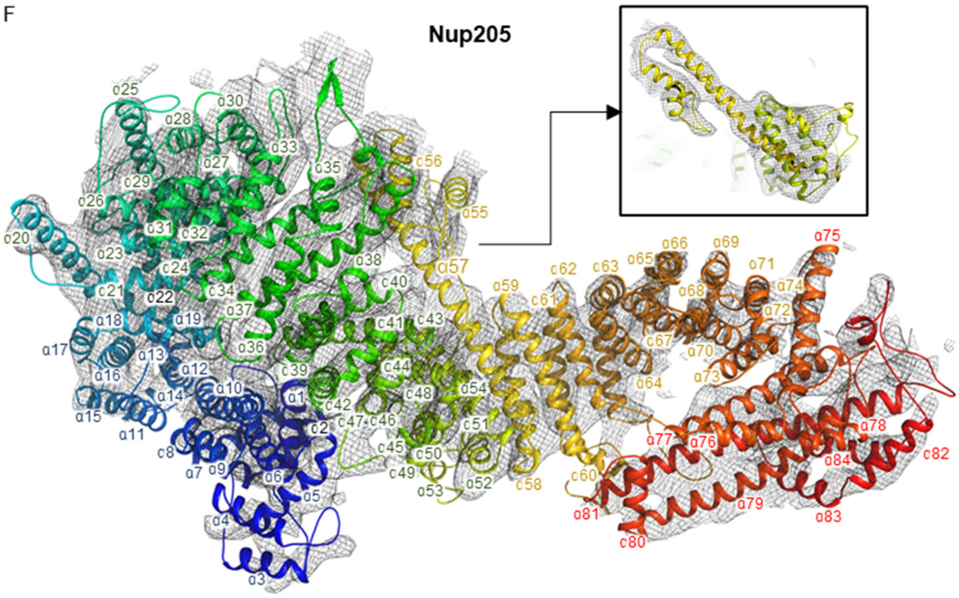

G

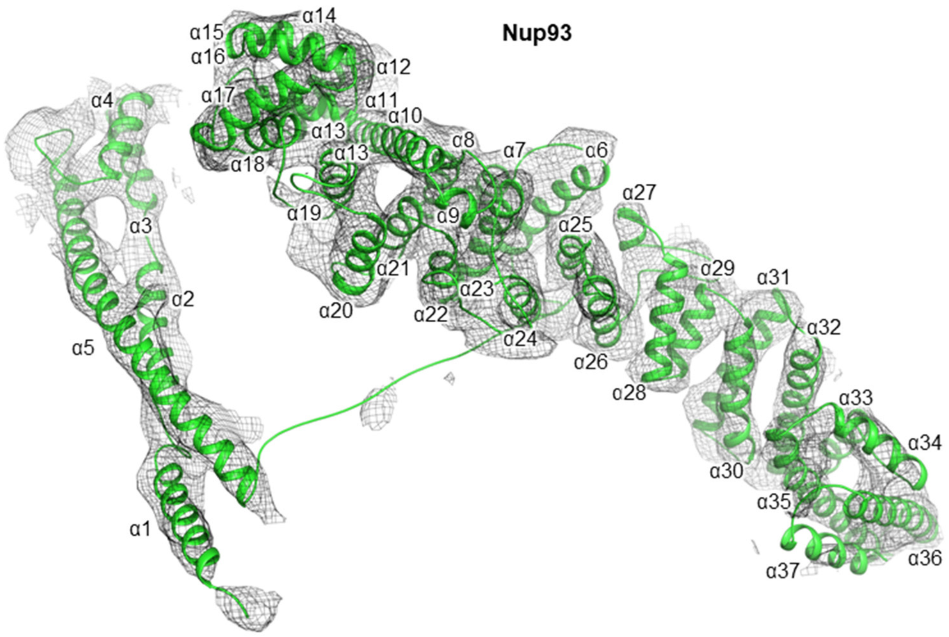

Figure S6. Model building quality and secondary structure assignment of the NR asymmetric unit. (A-G) Model building quality of Nup85, Seh1, Nup43, ELYS, Nup160, Sec13, Nup96, Nup133, Nup107, Nup205 and Nup93.

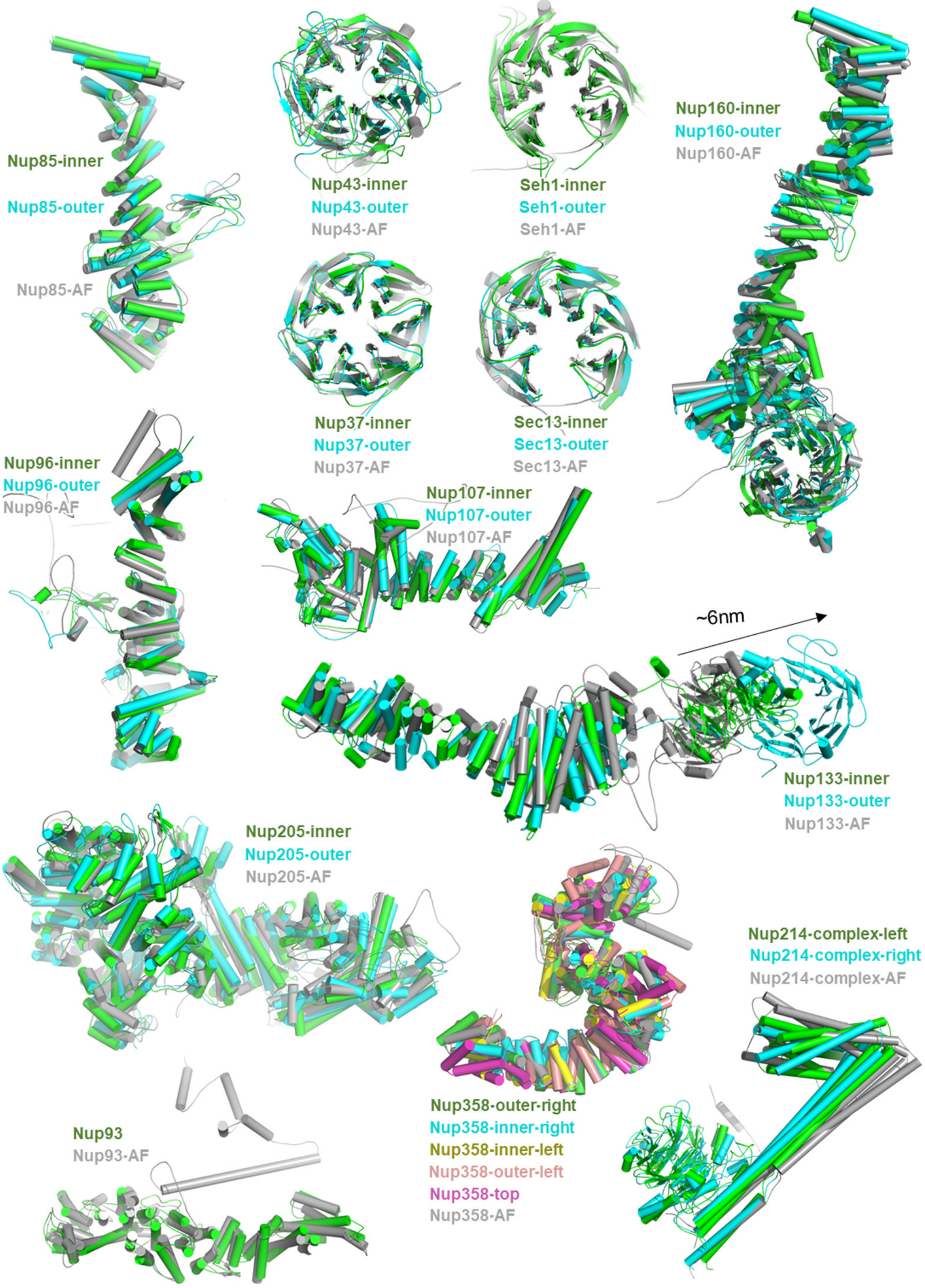

Figure S7. Comparisons of the refined structure and predicted structure by AlphaFold2 (AF) of CR Nups from the *X. laevis* NPC.

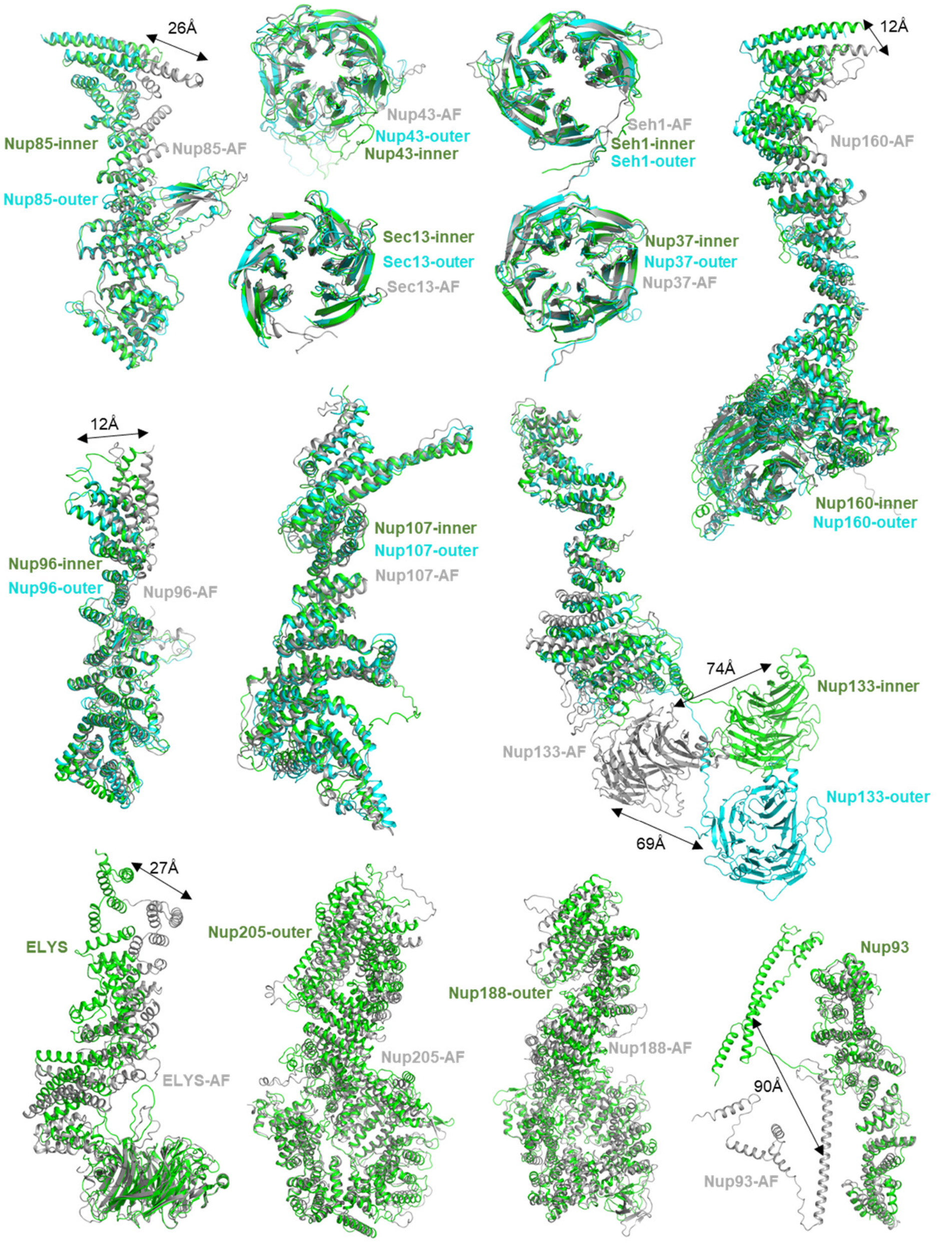

Figure S8. Comparisons of the refined structure and predicted structure by AlphaFold2 (AF) of NR Nups from the *X. laevis* NPC.

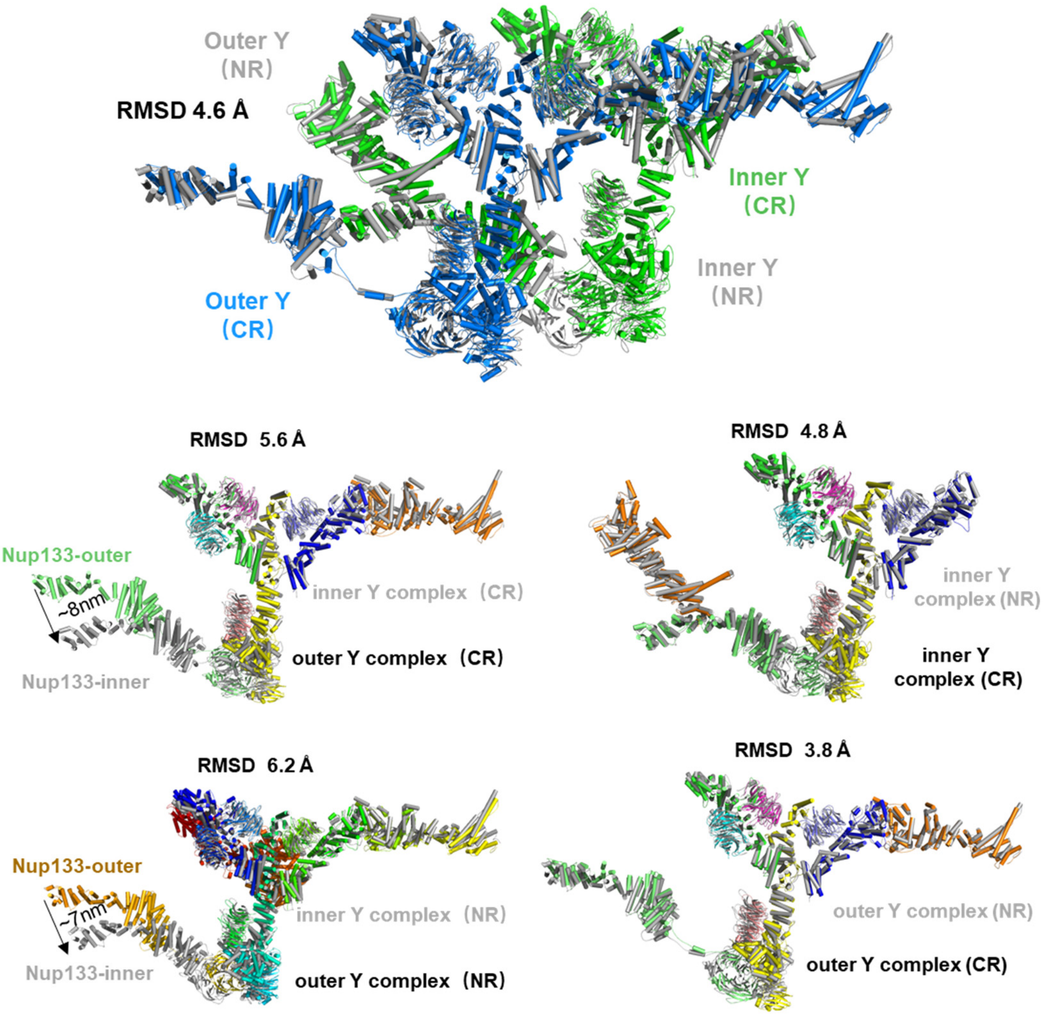

Figure S9. Structural comparations of Y-complexes in NR & CR from *X. laevis* NPC.

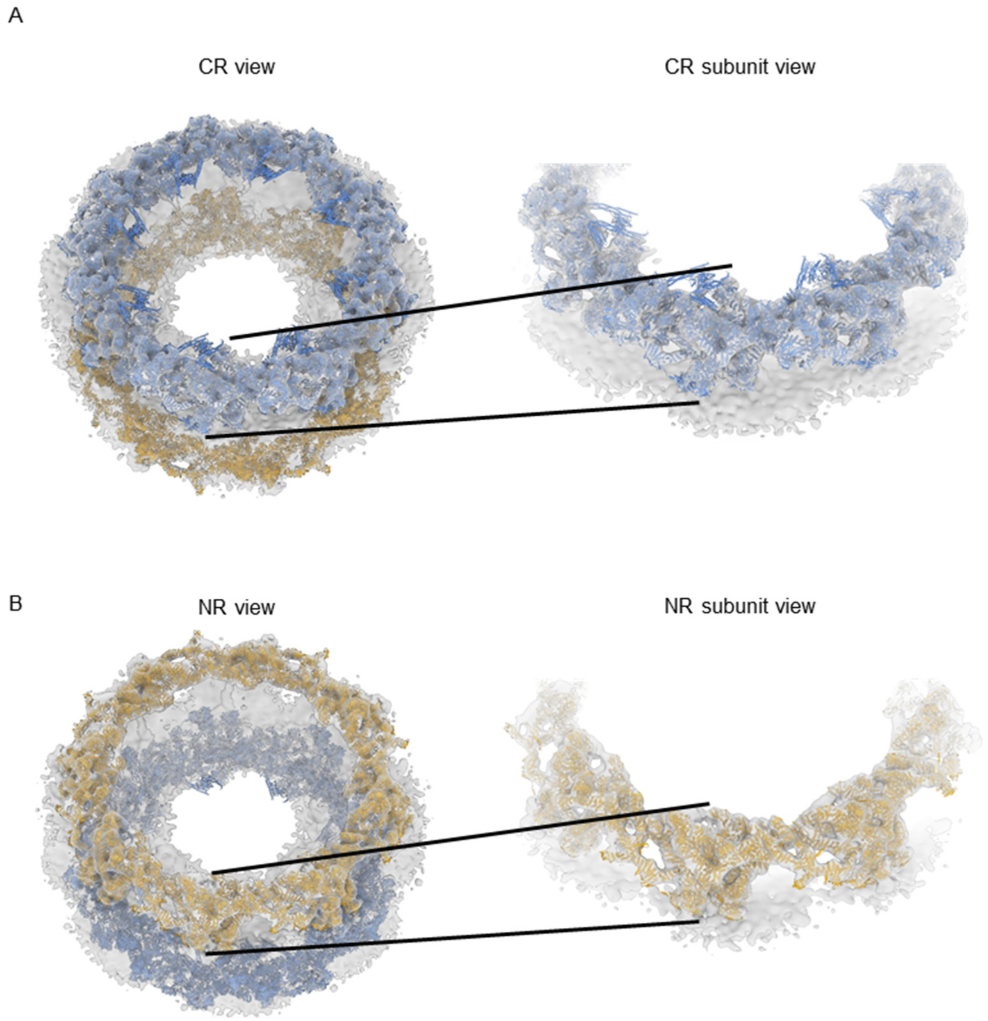

Figure S10. CR and NR models of *X. laevis* NPC fitted in human NPC map (EMD-3103).

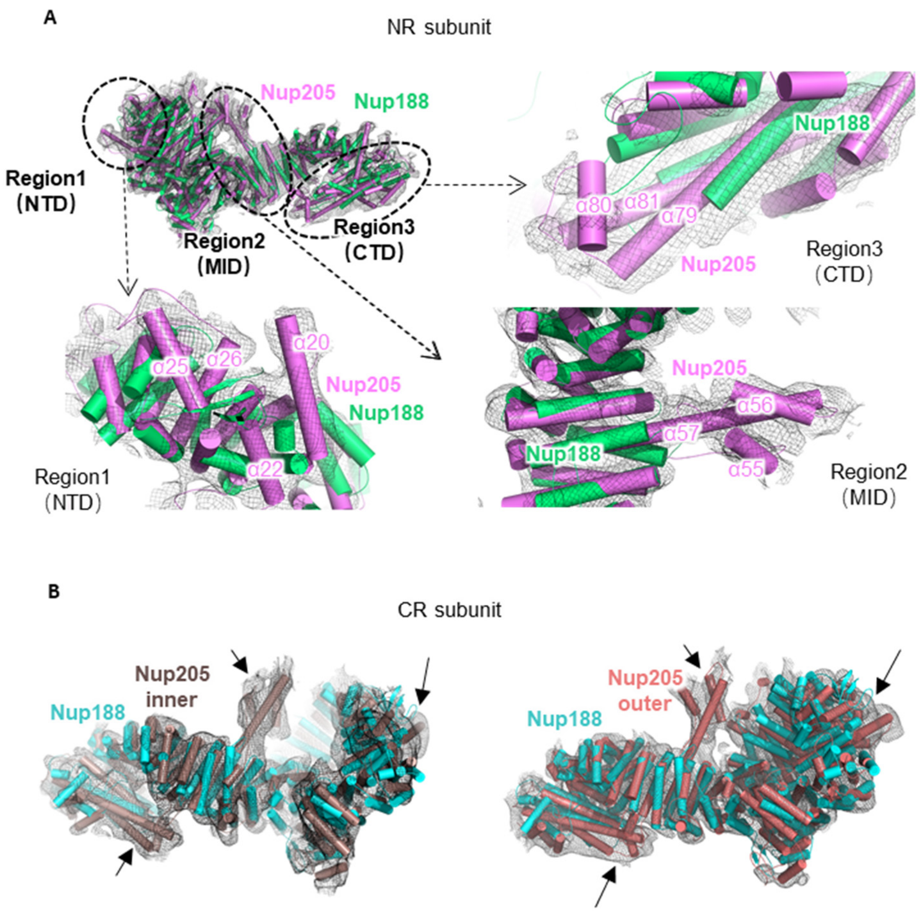

Figure S11. Nup205, instead of Nup188, is located in NR and CR. (A) Model comparison of Nup205 and Nup188 onto local densities in NR subunit. Major differences are enlarged and displayed in detail. (**B**) Model comparison of Nup205 and Nup188 onto local densities in CR subunit. The cryo-EM density maps are shown as a black mesh.

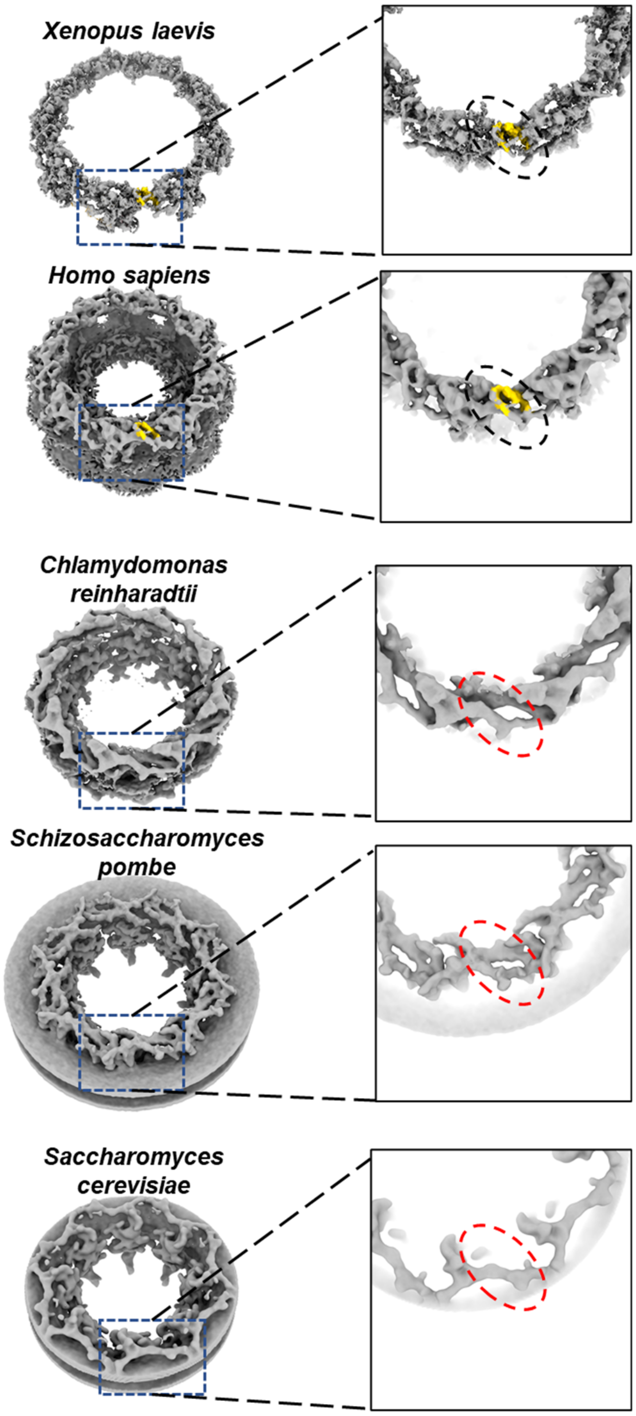

Figure S12. Comparisons of bridge-like densities in the NPC NR for different species.

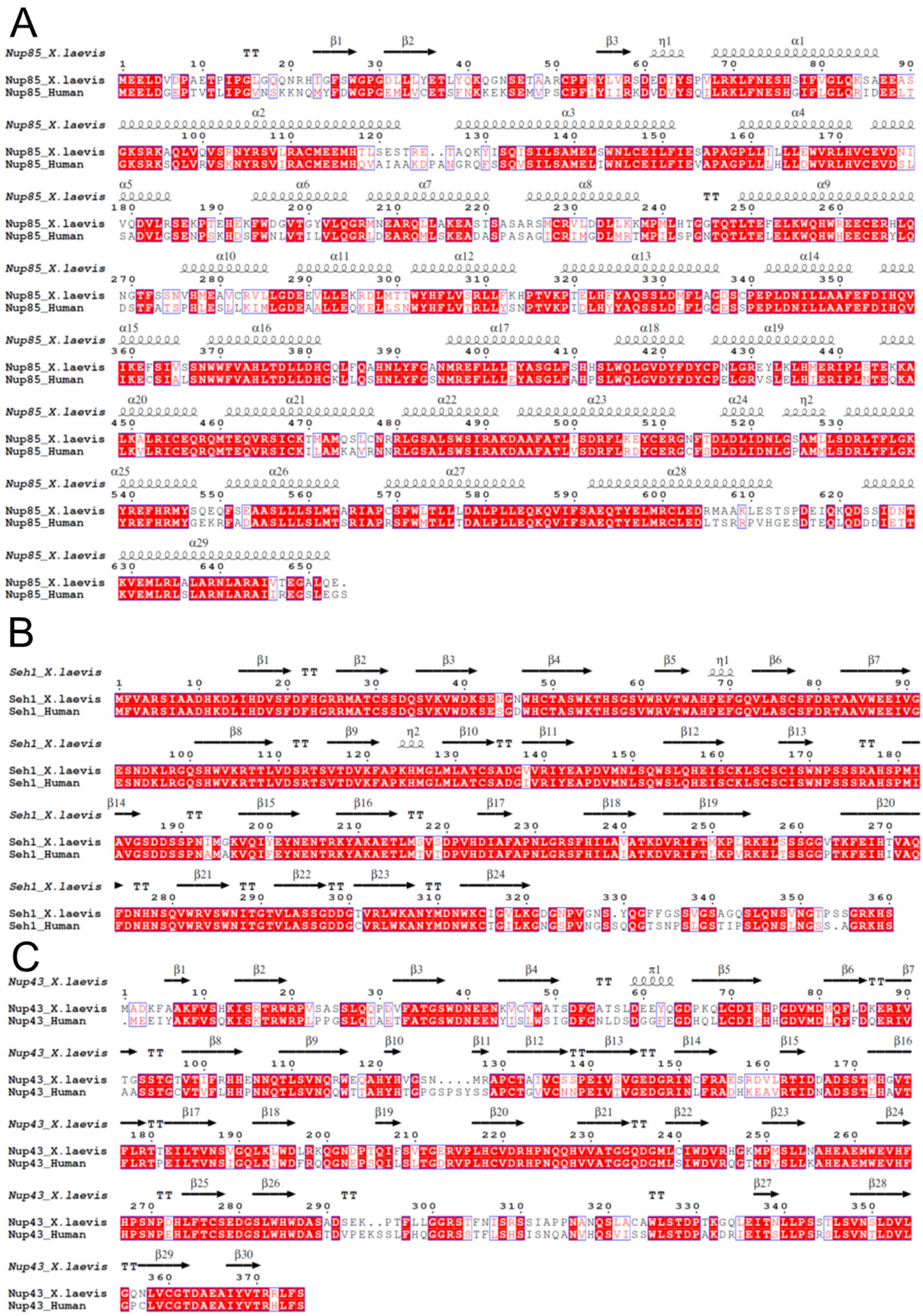
A
Nup85_X.laevis
Nup85_Human
B
Seh1_X.laevis
Seh1_Human
C
Nup43_X.laevis
Nup43_Human

D

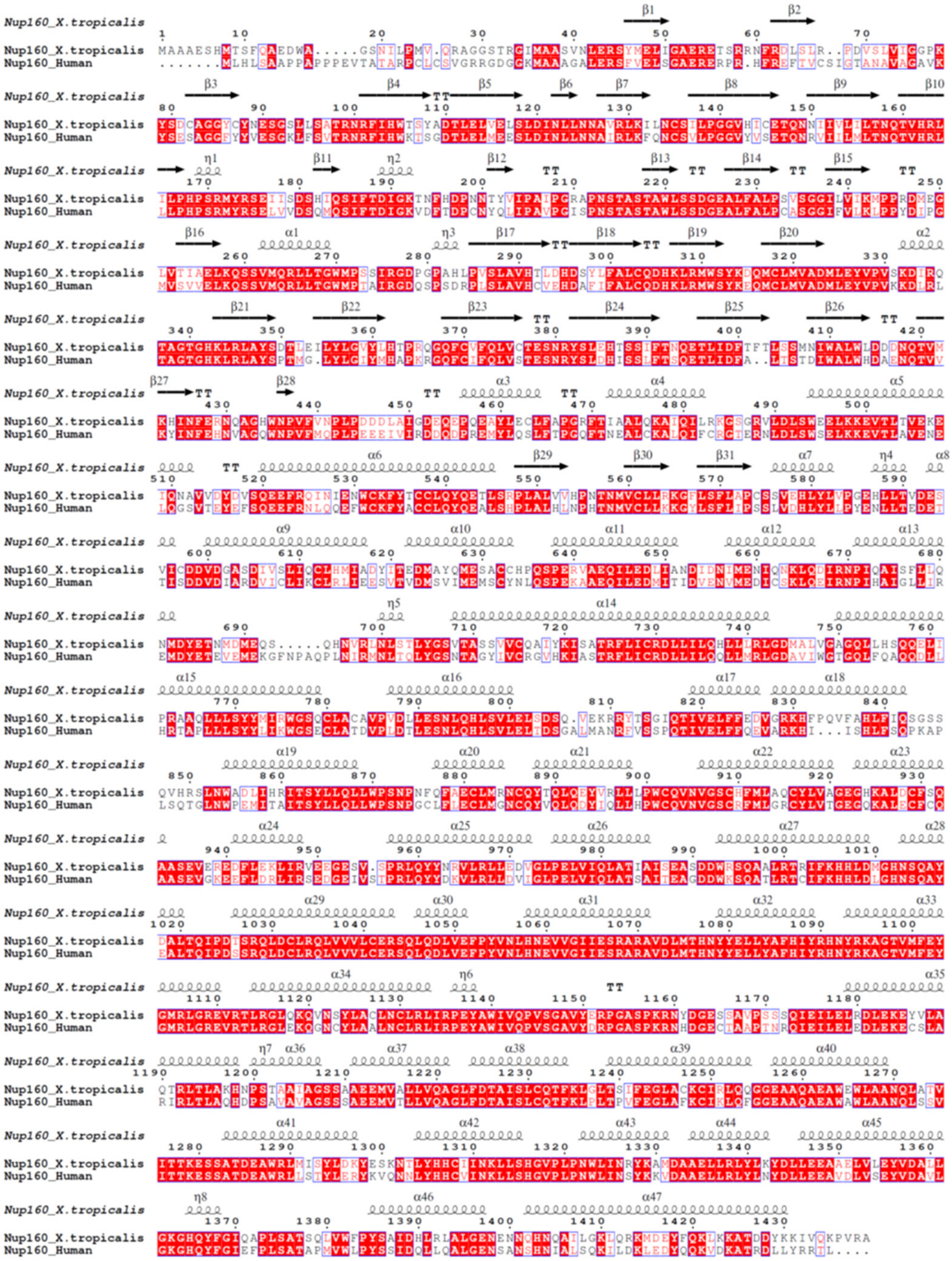

E

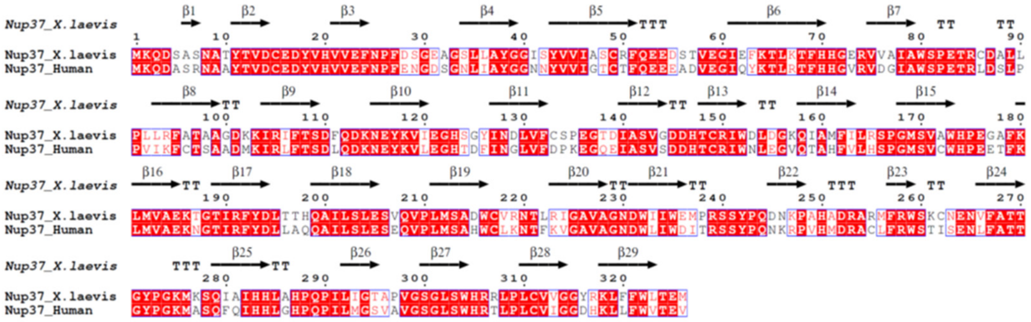
Nup37_X.laevis
Nup37_X.laevis
Nup37_Human

F

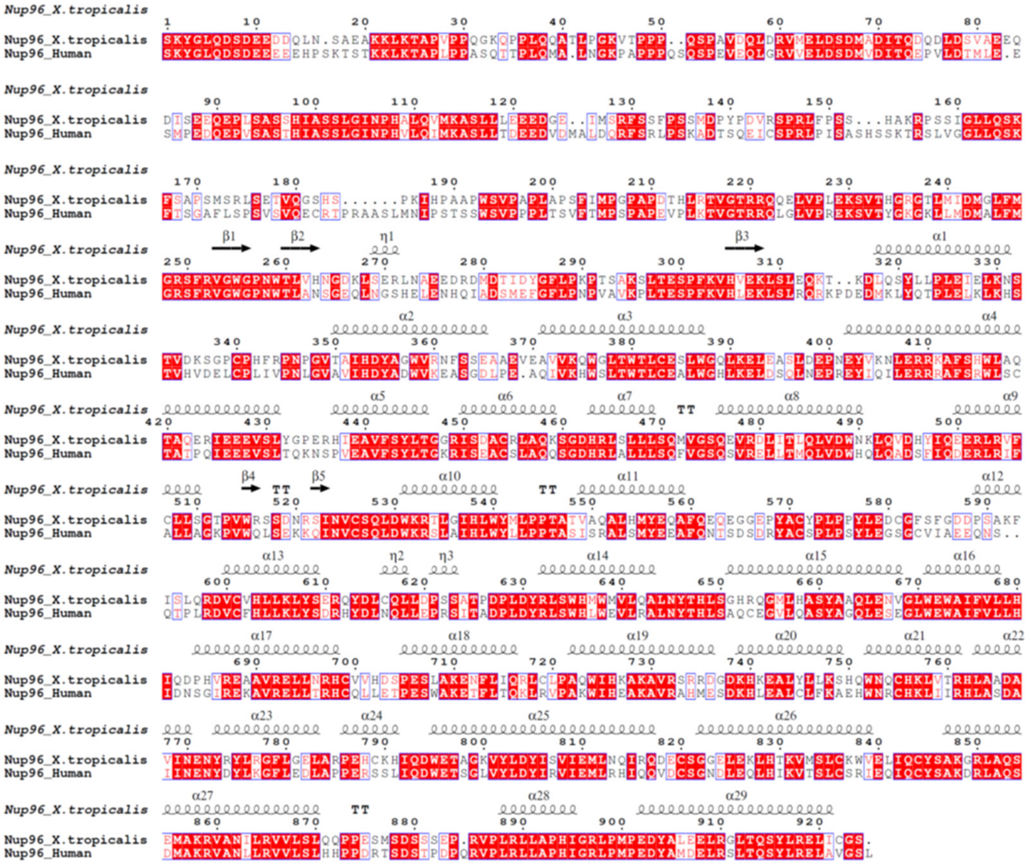
Nup96_X.tropicalis
Nup96_X.tropicalis
Nup96_Human

G

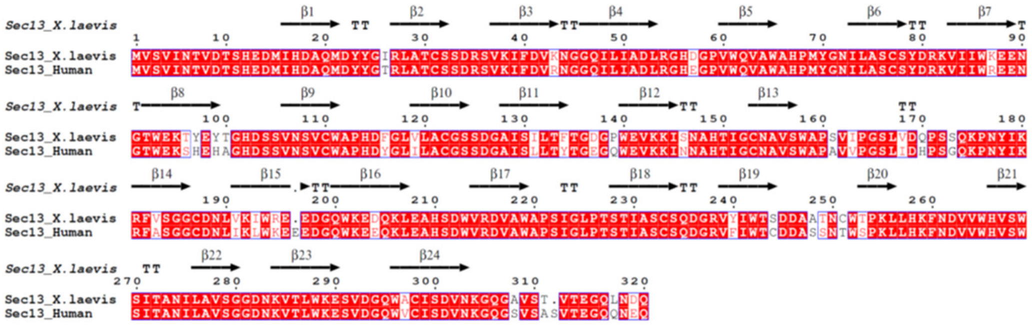
Sec13_X.laevis
Sec13_X.laevis
Sec13_Human

H

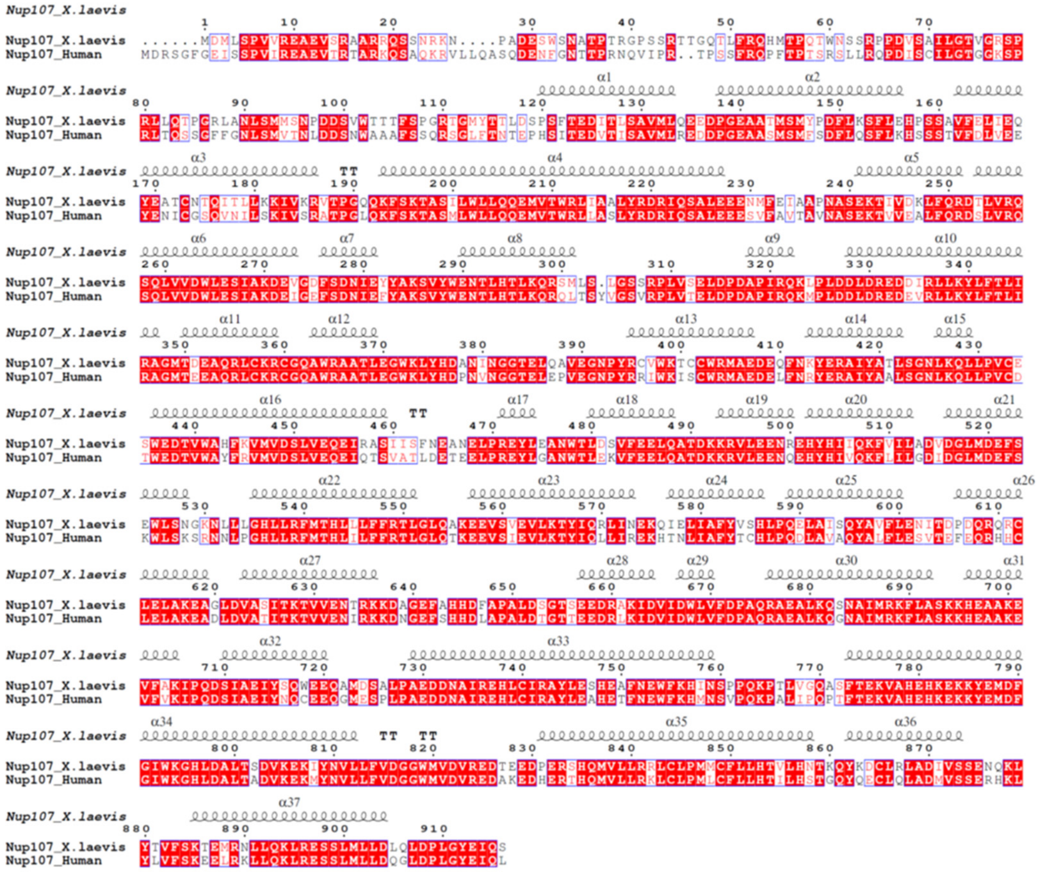
Nup107_X.laevis
Nup107_X.laevis
Nup107_Human

I

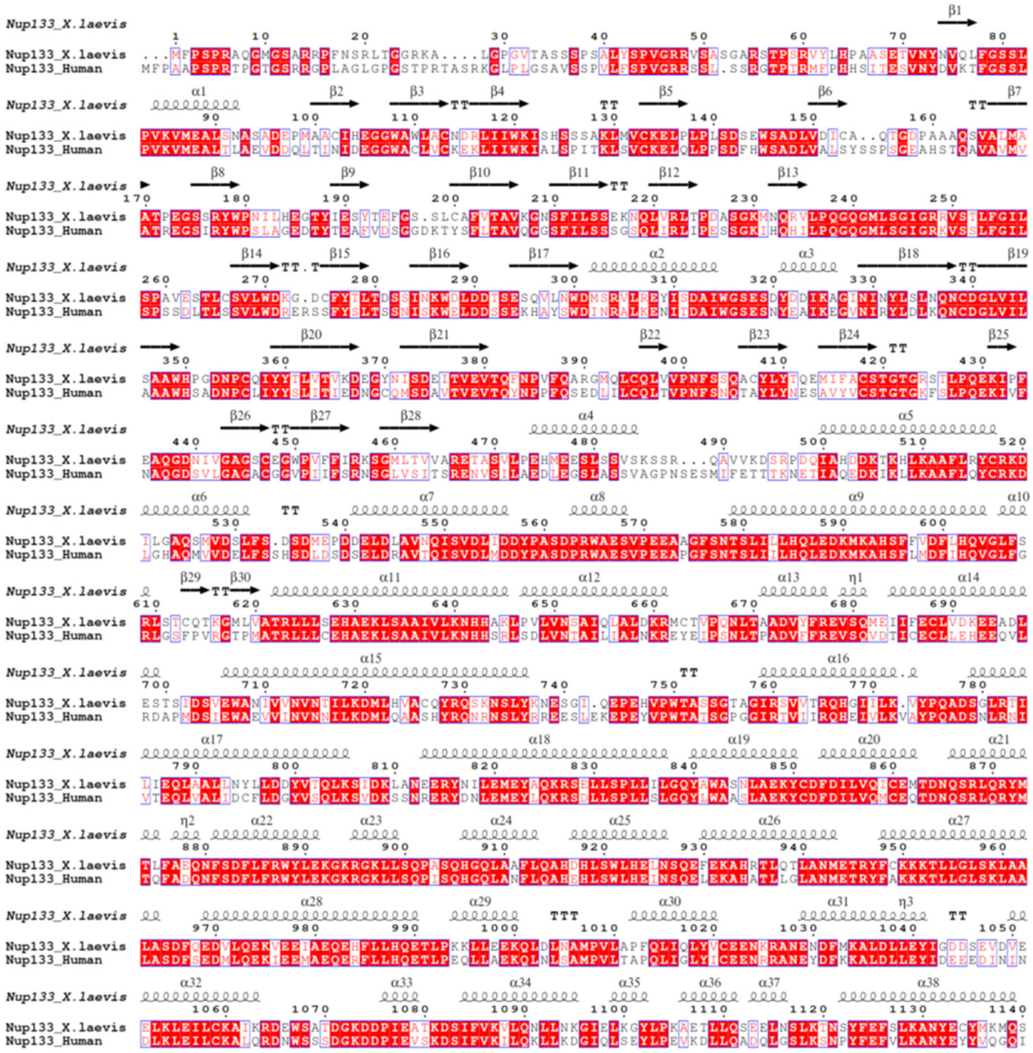

J

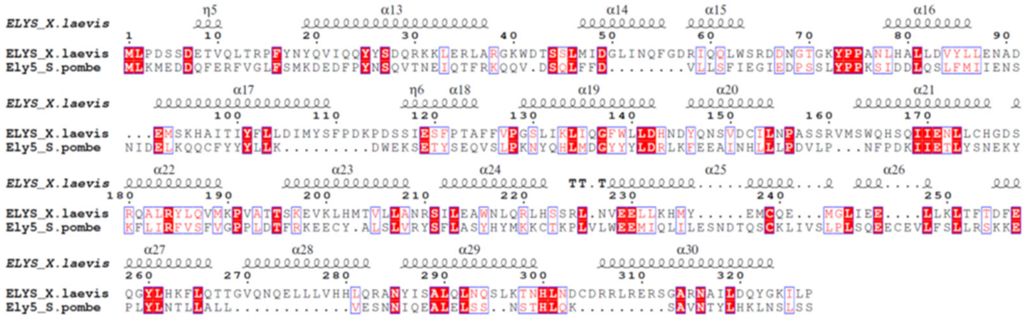

K

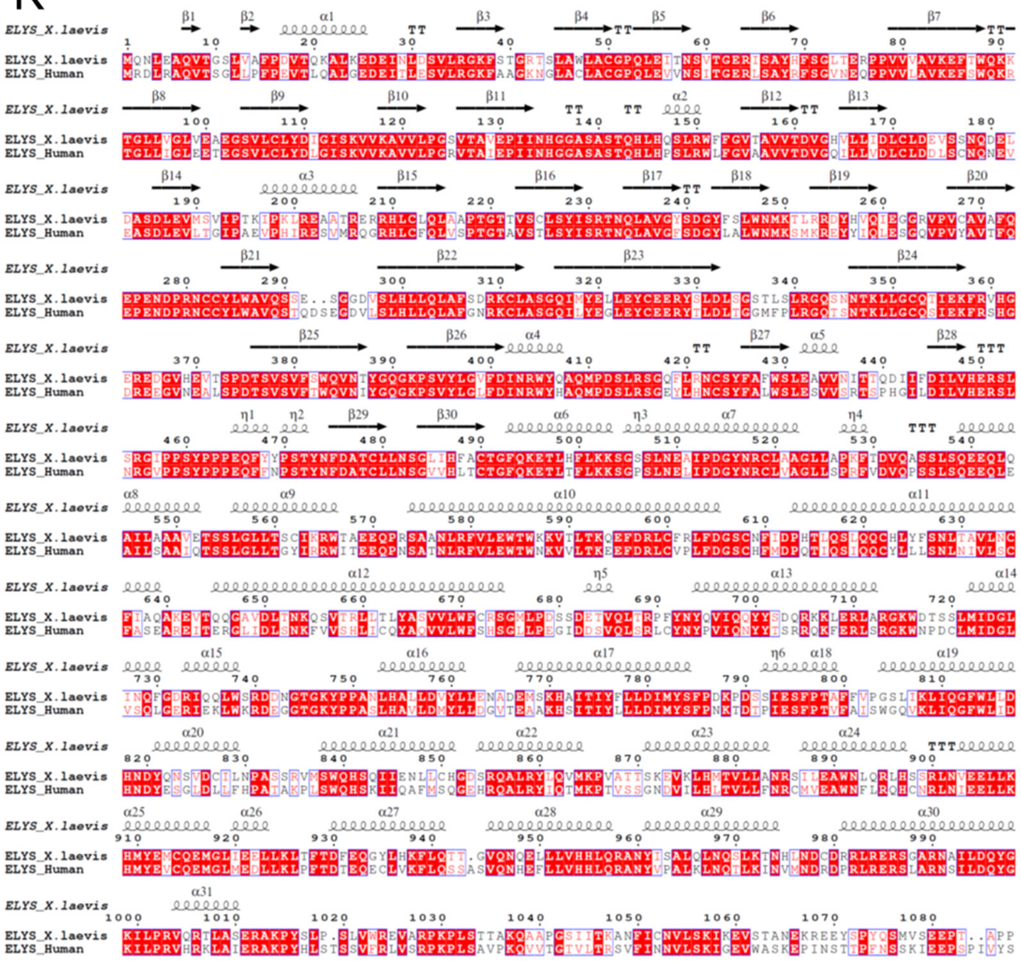

L

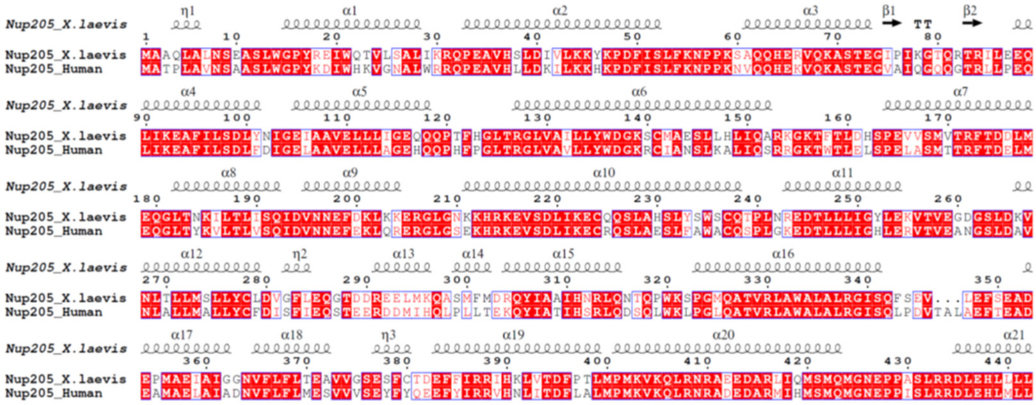

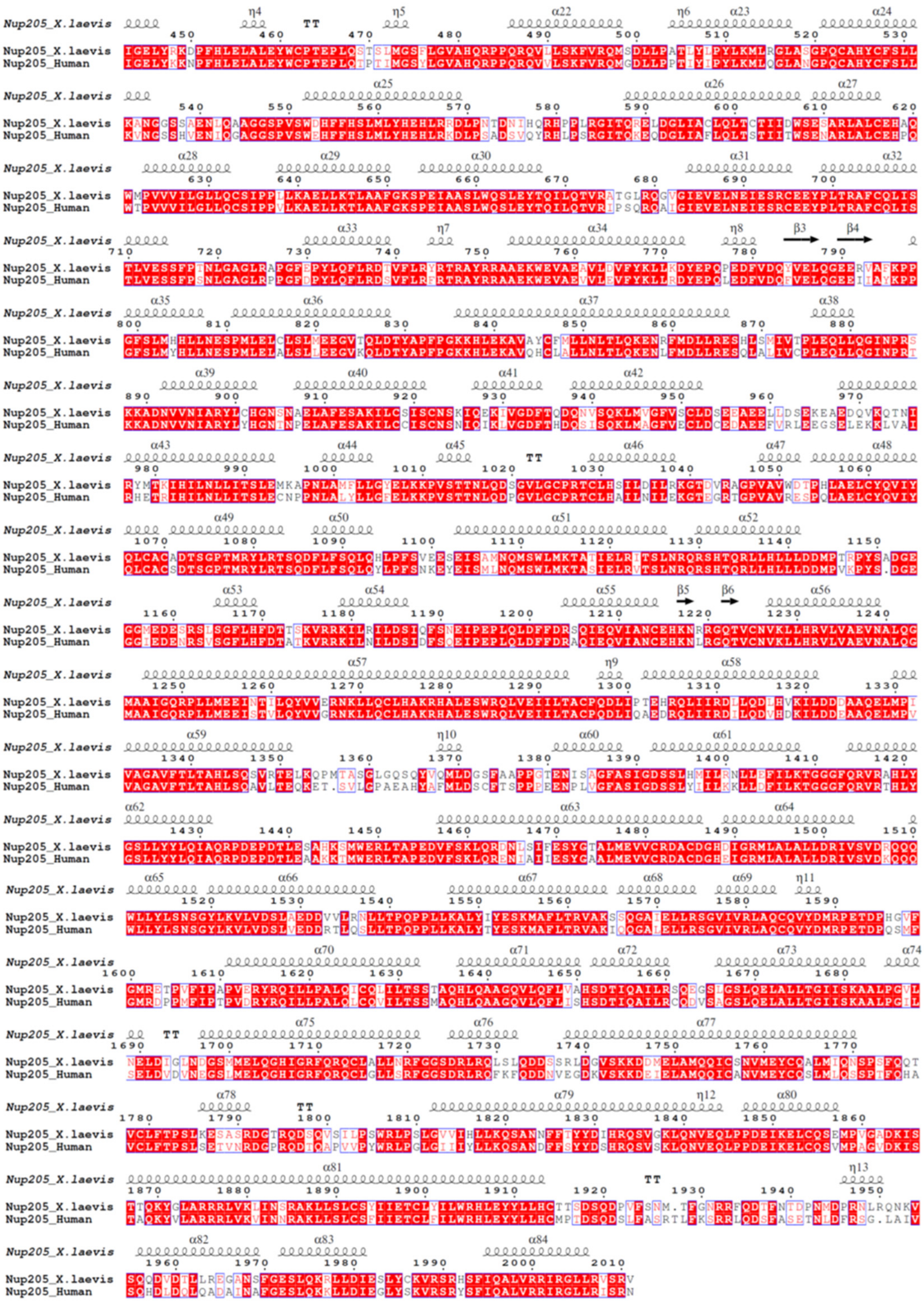
Nup205_X.laevis
Nup205_Human

M

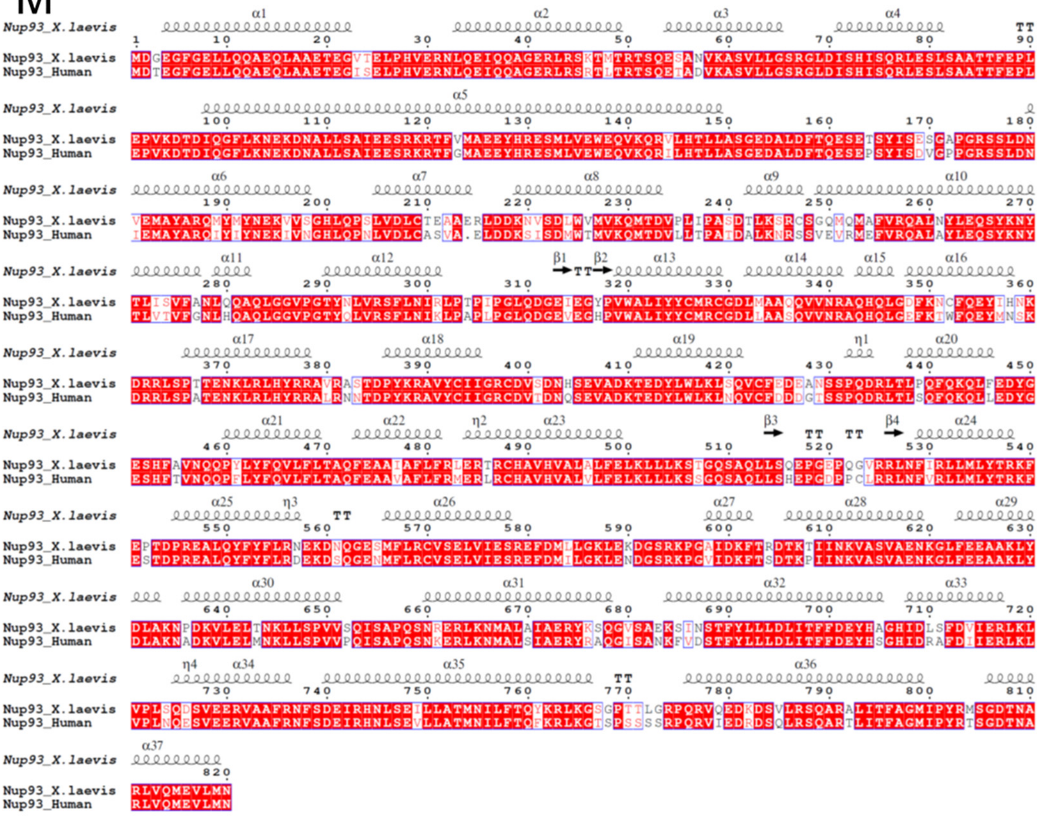
Nup93_X.laevis
Nup93_Human

N

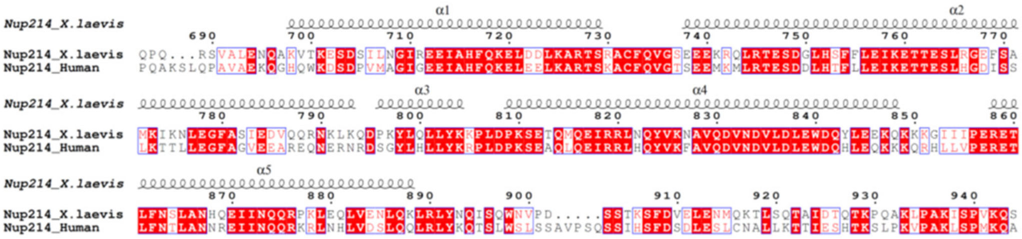
Nup214_X.laevis
Nup214_Human

O

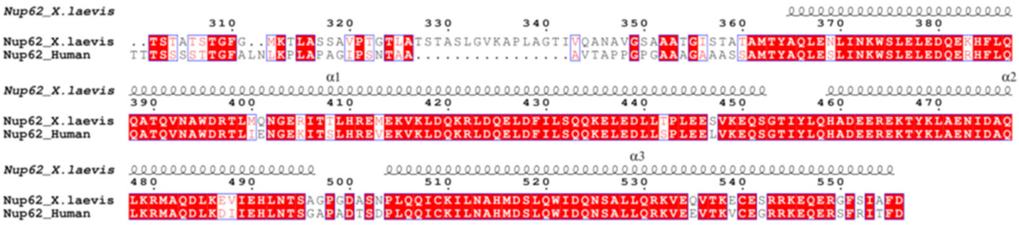
Nup62_X.laevis
Nup62_Human

P

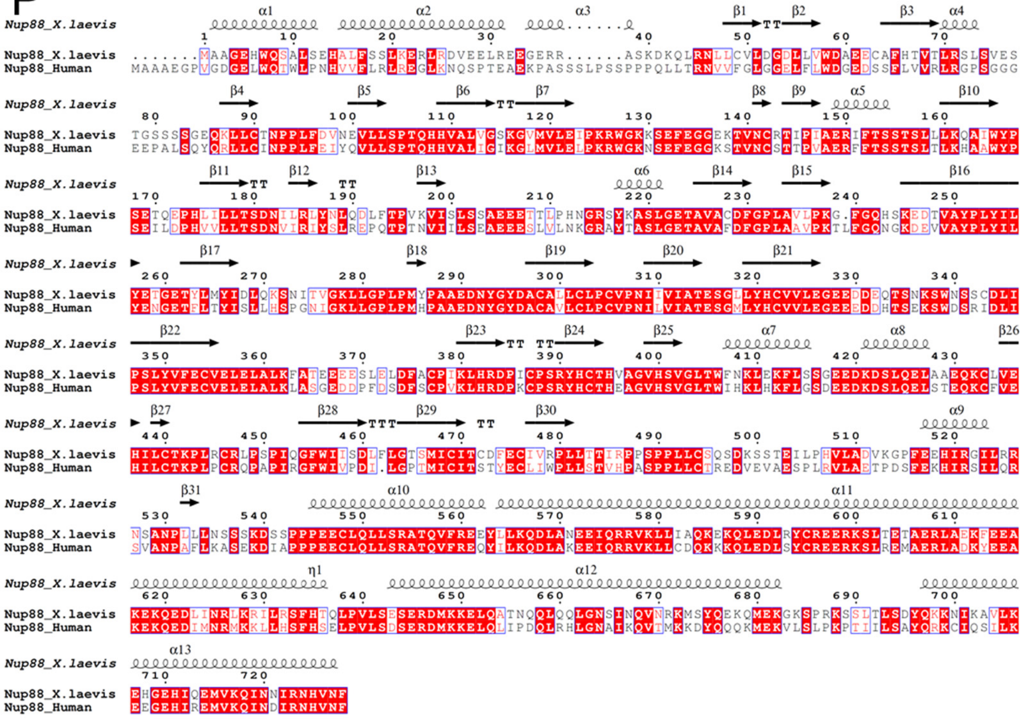

Q

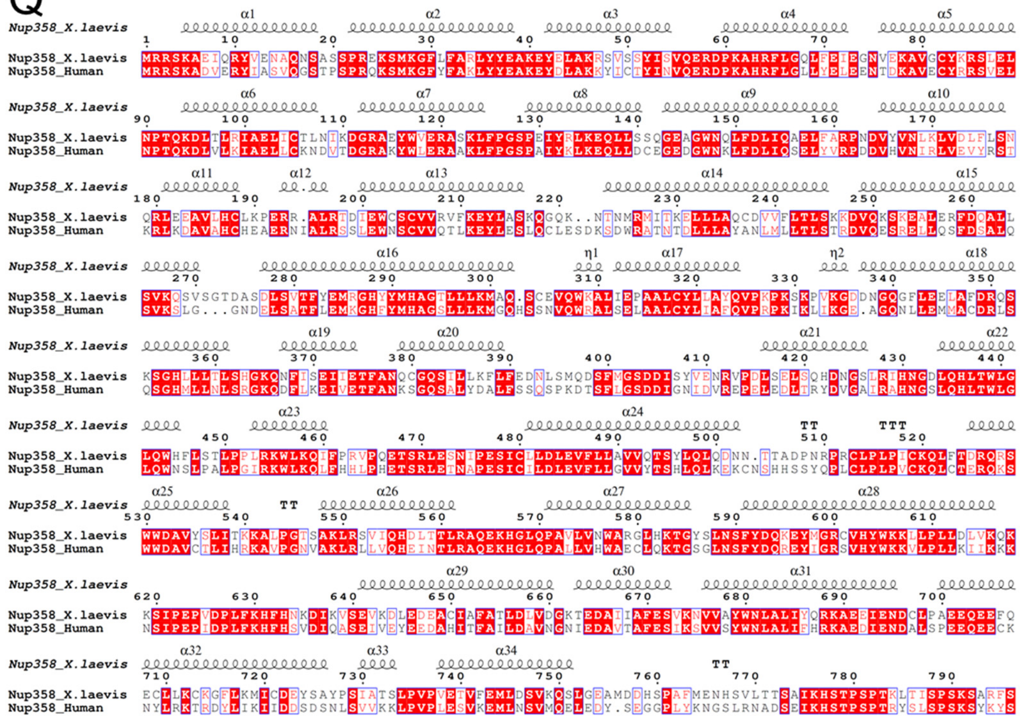

R

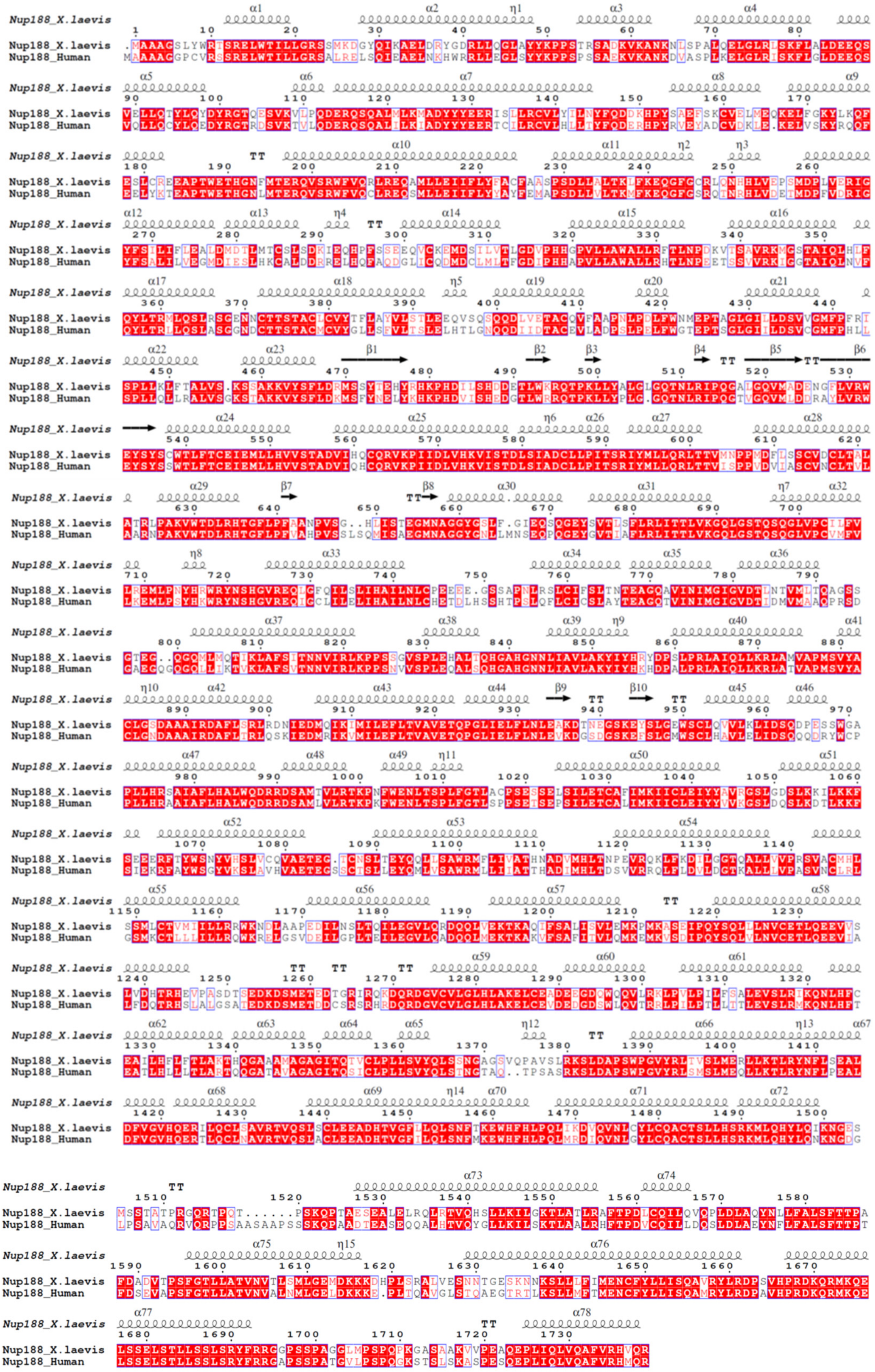

Figure S13. Sequence alignments of Nups in CR and NR from *X. laevis* (or *X. tropicalis*) and human NPCs.

Table S1 Statistics of cryo-SPA data collection and image processing.

**Data acquisition**			
Microscope	Titan Krios G2		
Voltage (kV)	300		
Detector	Gatan K2		
Energy filter	Gatan GIF Quantum, 20 eV		
Mode	Super resolution		
Pixel size (Å)	2.24		
Stage tilting angle	30°/ 45°/ 60°/ 0°(side-view)		
Exposure per tilt (e/Å²)	60 / 80/ 100/ 100 (or 120)		
Number of images	8745		
Defocus range (μm)	-1 ~ -4		
Software	SerialEM		
**Reconstruction in CR**			
Software	RELION-3.0/CryoSPARC		
Data set	CR core region	CR subunit region	CR Nup358 region
Final number of particles	354460	354460	678866
Symmetry	C1	C1	C1
Final resolution (Å)	8.0	8.7	8.9
Map pixel size (Å)	2.24	2.24	2.24
Map sharpening B-factor (Å²)	-591	-763	-850
**Reconstruction in NR**			
Software	RELION-3.0/CryoSPARC		
Data set	NR core region	NR subunit region	NR Nup133 region
Final number of particles	417490	417490	298318
Symmetry	C1	C1	C1
Final resolution (Å)	7.8	8.1	8.6
Map pixel size (Å)	2.24	2.24	2.24
Map sharpening B-factor (Å²)	-433	-541	-612

Supplementary Movies.

Movie S1. Overall structure of outer rings from the *X. laevis* NPC.

Movie S2. Model-map fitting quality of CR Nups from the *X. laevis* NPC.

Movie S4. Model-map fitting quality of NR Nups from the *X. laevis* NPC.

Movie S4. Structural comparison of Nup93 between the refined model and the model predicted by AlphaFold2.

Supplementary Scripts.

Script S1. The modified block-based reconstruction script for symmetry expanding of CR and NR asymmetric units.
